# The *MDM2* inducible promoter folds into four-tetrad antiparallel G-quadruplexes targetable to fight malignant liposarcoma

**DOI:** 10.1093/nar/gkaa1273

**Published:** 2021-01-07

**Authors:** Sara Lago, Matteo Nadai, Emanuela Ruggiero, Martina Tassinari, Maja Marušič, Beatrice Tosoni, Ilaria Frasson, Filippo M Cernilogar, Valentina Pirota, Filippo Doria, Janez Plavec, Gunnar Schotta, Sara N Richter

**Affiliations:** Department of Molecular Medicine, University of Padua, via A. Gabelli 63, 35121 Padua, Italy; Department of Molecular Medicine, University of Padua, via A. Gabelli 63, 35121 Padua, Italy; Department of Molecular Medicine, University of Padua, via A. Gabelli 63, 35121 Padua, Italy; Department of Molecular Medicine, University of Padua, via A. Gabelli 63, 35121 Padua, Italy; Slovenian NMR center, National Institute of Chemistry, Hajdrihova, 19, Ljubljana SI-1000, Slovenia; Department of Molecular Medicine, University of Padua, via A. Gabelli 63, 35121 Padua, Italy; Department of Molecular Medicine, University of Padua, via A. Gabelli 63, 35121 Padua, Italy; Division of Molecular Biology, Biomedical Center, Faculty of Medicine, LMU Munich, Germany; Department of Chemistry, University of Pavia, V. le Taramelli 10, 27100, Pavia, Italy; Department of Chemistry, University of Pavia, V. le Taramelli 10, 27100, Pavia, Italy; Slovenian NMR center, National Institute of Chemistry, Hajdrihova, 19, Ljubljana SI-1000, Slovenia; Division of Molecular Biology, Biomedical Center, Faculty of Medicine, LMU Munich, Germany; Department of Molecular Medicine, University of Padua, via A. Gabelli 63, 35121 Padua, Italy

## Abstract

Well-differentiated liposarcoma (WDLPS) is a malignant neoplasia hard to diagnose and treat. Its main molecular signature is amplification of the *MDM2-*containing genomic region. The *MDM2* oncogene is the master regulator of p53: its overexpression enhances p53 degradation and inhibits apoptosis, leading to the tumoral phenotype. Here, we show that the *MDM2* inducible promoter G-rich region folds into stable G-quadruplexes both *in vitro* and *in vivo* and it is specifically recognized by cellular helicases. Cell treatment with G-quadruplex-ligands reduces *MDM2* expression and p53 degradation, thus stimulating cancer cell cycle arrest and apoptosis. Structural characterization of the *MDM2* G-quadruplex revealed an extraordinarily stable, unique four-tetrad antiparallel dynamic conformation, amenable to selective targeting. These data indicate the feasibility of an out-of-the-box G-quadruplex-targeting approach to defeat WDLPS and all tumours where restoration of wild-type p53 is sought. They also point to G-quadruplex-dependent genomic instability as possible cause of *MDM2* expansion and WDLPS tumorigenesis.

## Graphical Abstract

**Graphical Abstract:**
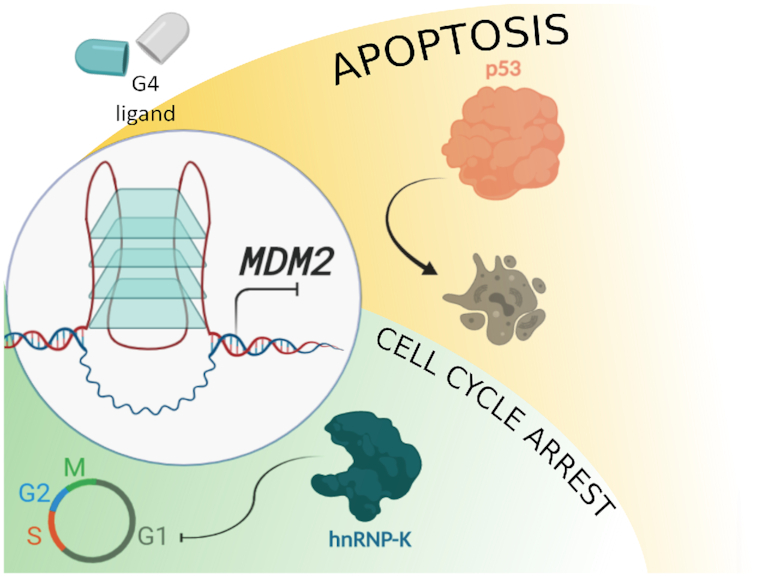
Scheme representing how G4-ligand-induced stabilization of MDM2 P2 promoter G4 leads to accumulation of p53 and hnRNP-K proteins, resulting in stimulation of apoptosis and cell cycle arrest.

## INTRODUCTION

Soft tissue sarcomas (STS) are rare malignant tumours that arise in any of the mesodermal tissues of the extremities (50%), trunk and retroperitoneum (40%), head and neck (10%). Among STS, liposarcomas (LPSs) are the most common sarcoma subtypes: they are neoplasms of adipocytes that normally grow slowly and present as a painless non-ulcerated enlarging mass (1). The World Health Organization classifies LPSs into: (i) well-differentiated/atypical lipomatous tumour (WDLPS); (ii) dedifferentiated LS (DDLPS); (iii) myxoid (round cell) LPS; (iv) pleomorphic LPS; (v) LPS, not otherwise specified (2). WDLPS represents 40–45% of the LPSs and is mainly treated with surgical resection (3). However, lesions in the retroperitoneum and inguinal areas are difficult to treat and tend to recur locally and transform into the more aggressive DDLPS. If the tumour is unresectable or if the excision was incomplete, systemic therapies and radiation can be proposed. However, both WDLPS and DDLPS respond very poorly to conventional chemotherapy (4–6).

In 90% of cases, WD/DDLPSs are characterized by extensive chromosomal aberrations, including amplification of the chromosomal region 12q13-15. This amplification causes overexpression of genes that promote cell growth, i.e. *MDM2*, *CDK4* and *HMGA2* (3).

*MDM2* is a well-characterized oncogene, involved in the autoregulatory negative feedback loop that modulates p53 tumour suppressor protein. The latter binds to the inducible promoter of *MDM2* and stimulates its transcription. In turn, accumulated MDM2 is responsible for p53 ubiquitination and consequent proteasomal degradation (7). Inhibition of the MDM2-p53 interaction to restore p53 activity is an appealing strategy to treat all cancers that overexpress MDM2 and/or have wild type p53. In fact, inhibitors of the MDM2-p53 interaction have been tested in clinical trials in WD/DDLPS patients. Imidazoline derivatives, termed nutlins, have shown tumour inhibition or regression, balanced however by limited response rates and, in some cases, serious adverse effects due to haematological toxicity (8,9).

*MDM2* transcription is regulated at two promoters: P1, located upstream of exon 1, active at the basal constitutive level and p53-independent; P2, located within intron 1 and p53-responsive (10). Transcription from P1 promoter is eight-fold less efficient than that from P2 (11), thus P2 promoter is the main responsible for *MDM2* mRNA accumulation. P2 promoter is extremely rich in guanines (65% GC-content) and it displays several binding sites for transcription factors, such as Sp1, MYCN, AP1, ETS and p53, suggesting tight regulation of its activation (12).

DNA guanine (G)-rich sequences are prone to fold into G-quadruplexes (G4s), non-canonical secondary structures. G4s are four-stranded helical structures formed in nucleic acids with runs of two or more guanines (Gs). Hoogsteen hydrogen bonds between Gs induce formation of planar G-quartets, stacked one upon the other and stabilized by physiological monovalent cations, such as K^+^. Despite the common G-quartet core, G4s are topologically polymorphic, as they can assemble into multiple conformations (13). From a functional point of view, the importance of G4s in the regulation of multiple biological processes *in vivo* is clearly emerging (14). G4s are highly represented in human oncogene promoters and telomeres and their involvement in carcinogenesis is being widely investigated (15). Moreover, G4 formation in repeated regions is associated to genome instability (16). Several G4-ligands with the ability to reduce or suppress oncogene expression upon G4 stabilization have been developed (17).

To improve the anticancer effects of MDM2 repression, we envisaged a DNA-targeting approach to repress MDM2 expression at the transcriptional level. We reasoned that if G4 structures are present in the *MDM2* P2 promoter, G4-ligands could be exploited to modulate *MDM2* transcription, reconstituting the p53 pro-apoptotic signal. We here identified peculiarly folded and highly stable G4s in the *MDM2* P2 promoter, proved their presence in the cellular environment and monitored the downstream effects of their targeting with a G4-ligand, which decreased MDM2 expression and recapitulated p53 levels with consequent apoptotic and cell cycle arrest effects.

## MATERIALS AND METHODS

### Cell lines, oligonucleotides and compounds

The 93T449 cell line (ATCC #CRL-3043) was cultured in RPMI medium (Gibco, Thermo Fisher Scientific, Waltham, MA, USA) supplemented with 10% FBS. Human normal lung fibroblasts (HNLF) were kindly provided by Dr Marco Montagner (University of Padua, Italy) and cultured in DMEM medium (Gibco, Thermo Fisher Scientific, Waltham, MA, USA) supplemented with 10% FBS. Desalted oligonucleotides were purchased from Sigma-Aldrich (Milan, Italy). A detailed list of the oligonucleotides’ names and sequences is available in Supplementary Table S1. Compound c-exNDI was synthesized as previously described (18,19), Nutlin-3a (20) was purchased from Sigma Aldrich (cat. no. SML0580, Milan, Italy), Quarfloxin was kindly provided by TetraGene, LLC (Salt Lake City, UT, USA).

### Prediction of putative G-quadruplexes

The *MDM2* first intron (GRCh38.p2, NC_000012.12) was analysed by QGRS Mapper (http://bioinformatics.ramapo.edu/QGRS/index.php) for the prediction of G4 forming sequences according to the motif G*x*N*y*1G*x*N*y*2G*x*N*y*3G*x*, where *x* is the number of guanine (G) tetrads, N is any deoxynucleotide and y the length of loops connecting the G tetrads. The following restrictions were applied: (i) the number of tetrads had to be ≥2; (ii) the maximum length of pG4s was set to 40 bases; (iii) the loop size was allowed to be 0–15 nt-long; (iv) only one loop was allowed to have *y* = 0. The found pG4s were ranked based on the G-score, which is the likelihood to form a stable G4, according to the following principles: (a) shorter loops are more common than longer loops; (b) G4s tend to have loops roughly equal in size; (c) the greater the number of G-quartets, the more stable the G4.

### PCR stop assay

Genomic DNA of 93T449 cells was extracted and purified using the Quick-DNA Universal Kit (Zymo Research #ZRC186999). 100 pmol of the six reverse primers spanning the whole *MDM2* P2 promoter (Supplementary Table S1) were 5′-end-labeled with [γ-^32^P]ATP by T4 polynucleotide kinase for 30 min at 37°C and purified with MicroSpin G-25 columns (GE Healthcare Europe, Milan, Italy). PCR amplification of *MDM2* gene was carried out with or without 200 nM G4-ligands (c-exNDI and Quarfloxin), in the presence of 15 pmol radiolabelled reverse primer and complementary cold primer, with the following cycling conditions: 1 × 5 min 95°C, 35 × 30 s 95°C – 45 s 54°C – 60 s 72°C, 1 × 7 min 72°C. PCR products were purified with Nucleospin Gel and PCR Clean Up (Macherey-Nagel #740609). Markers G+A, produced by Maxam and Gilbert treatment, and the intact reverse primer and PCR product were loaded onto a 8% denutauring polyacrylamide gels containing 8 M urea and visualized by phosphorimaging analysis on a Typhoon FLA 9000 (GE Healthcare Europe, Milan, Italy). Quantification of stop bands was performed using ImageQuant software and calculated as percentage of the full-length amplification product.

### BG4 purification, G4-immunoprecipitation and qPCR

The FLAG-tagged anti-G4 antibody BG4 was produced from the BG4-encoding plasmid pSANG10-3F-BG4 (kindly provided by Prof. S. Balasubramanian, University of Cambridge, UK). BL21(DE3) competent cells (Stratagene) were cultured in TY medium (1.6% tryptone peptone, 1% yeast extract and 0.5% NaCl) and 50 μg/ml kanamycin, transformed with the plasmid and induced with 0.85 mM isopropyl β-d-1-thiogalactopyranoside. The cells were lysed (20 mM Tris–Cl pH 8.0, 50 mM NaCl, 5% glycerol, 1% Triton and 100 μM phenylmethanesulfonylfluoride solution) by repeated freezing/thawing. The supernatant was filtered (0.45 μm) and purified on a Protino Ni-NTA-Agarose Affinity column (Machery-Nagel, Germany) according to the manufacturer instructions. BG4 antibody was concentrated in Amicon Ultra-3k Centrifugal Filter Unit (Millipore). For G4-immunoprecipitation procedure, 93T449 cells were fixed in 1% formaldehyde for 10 min and quenched with 0.125 M glycine. Chromatin was sonicated using the Covaris E220 to shear to an average size of 100–500 bp, digested with DNase-free RNase (Thermo Scientific, cat. EN0531) and incubated in the presence of BG4. Anti-FLAG antibody (Sigma-Aldrich, Milan, Italy, cat. F-3165) coated Protein-G magnetic beads (Pierce™ ThermoFisher Scientific) were used to capture BG4-G4 complexes. After de-crosslinking, samples were purified with the MinElute PCR Purification Kit (Qiagen, cat. 28006). G4 enrichment was quantified via qPCR, using Fast SYBR PCR mix (Applied Biosystems), with a LightCycler 480 (Roche) quantitative PCR machine. Primer pairs that target G4 ChIP positive and negative regions were employed (Supplementary Table S1). Relative enrichments were derived with respect to their inputs.

### Protein pull-down and LC–MS identification

93T449 nuclear extracts were obtained with NXTRACT kit (Sigma-Aldrich, Milan, Italy) and quantified using Pierce™ BCA Protein Assay Kit (Thermo Fisher Scientific, Monza, Italy). Biotinylated oligonucleotides (Sigma-Aldrich, Milan, Italy) were diluted to 3 μM in phosphate buffer pH 7.4 20 mM supplemented with KCl 100 μM (PB–KCl buffer), heat denatured for 5 min at 95°C and slowly cooled to RT to allow secondary structures folding. Streptavidin coated magnetic beads (Dynabeads^®^ M-280 Streptavidin, Thermo Fisher Scientific, Monza, Italy) were functionalized with 150 pmol of folded oligonucleotides (*Mdm2-biot*, *G-ss-biot* as a single stranded G-rich control and *C-ss-biot* as C-rich single stranded control) or PB–KCl buffer only as negative control. 25 μg nuclear extracts were added and incubated at 4°C for 2–3 h. Specific bound proteins were first eluted in NaCl 2 M and then in 2× Laemmli buffer (4% SDS, 80% glycerol, 120 mM Tris–HCl pH 6.8, 200 mM DTT, 0.02% bromophenol blue). Eluted proteins were separated on a 8% SDS-PAGE and in-gel digested as previously described (21). The peptide mixture was analysed by LC–MS, using a low pressure Acquity H-class bioQuaternary Solvent Manager UPLC (Waters, Manchester, UK) system, a Jupiter proteo^®^ RP12 (1.0 × 150 mm, 4 μm, 90 Å) (Phenomenex, Torrance, CA, USA) chromatographic column and mass analyser Xevo G2-XS QTof mass spectrometer (Waters, Manchester, UK). Proteins were identified from partent ions by Mascot Database Search (http://www.matrixscience.com).

### Liquid chromatography–mass spectrometry (LC–MS)

G4 oligonucleotide LC–MS analysis was performed as previously described (22). Briefly, oligonucleotides diluted to 5 μM in 2.5 mM KCl (Fluka, St. Louis, MO, USA), 120 mM trimethylammonium acetate (TMAA) (Fluka) pH 7.4 (adjusted from pH ∼7 to 7.4 with triethylamine (TEA) (Fisher, Pittsburgh, PA, USA)) were annealed by heating at 95°C for 5 min, gradually cooled to room temperature. Samples were analyzed by direct infusion electrospray ionization (ESI) on a Xevo G2-XS QTOF mass spectrometer (Waters, Manchester, UK). The injection was performed by an Agilent 1290 Infinity HPLC (Agilent Technologies, Santa Clara, CA, USA) in TMAA 120 mM pH 7.4. ESI source settings were: electrospray capillary voltage 1.8 kV; source and desolvation temperatures 45 and 65°C, respectively; sampling cone voltage 85 V. LC–MS spectra deconvolution was performed either manually and by automatic calculation using MassLynx and BioPharmaLynx softwares. Oligonucleotide exact mass was calculated by mean of the MongoOligo tool (https://mods.rna.albany.edu/masspec/Mongo-Oligo). Manual deconvolution was calculated from the parental peak of single charge states of the oligo as: *m*/*z* × charge state + charge state, the obtained mass was compared to the exact oligonucleotide mass plus the corresponding number of K^+^ ions, where a single K^+^ ion has a mass of 39.0983 Da. Automatic deconvolution was performed on molecules, the signal of which was detected in a time window of typically 1–3 min after sample injection; the *m*/*z* range on which deconvolution was performed was set in order to comprise all the visible peaks corresponding to the input sample: 600–3000 *m*/*z*; the molecular weight (MW) to be analyzed was set to the input oligonucleotide exact mass ± 1000 Da. The obtained list of masses was then compared to the exact oligonucleotide mass plus the corresponding number of K^+^ ions.

### Dimethylsulphate footprinting assay

The DNA substrate of interest was purified before use by running it on a 20% denaturing polyacrylamide gel and eluted in water solution overnight at RT shaking. Eluates were concentrated by using Amicon Ultra-3k Centrifugal Filter Unit (Millipore). The purified oligonucleotides were 5′-end-labeled with [γ-^32^P]ATP by T4 polynucleotide kinase for 30 min at 37°C, purified using MicroSpin G-25 columns (GE Healthcare Europe, Milan, Italy), resuspended in lithium cacodylate buffer 10 mM pH 7.4 with or without KCl 100 mM, heat-denatured for 5 min at 95°C and folded at RT overnight. Sample solutions were then treated with dimethylsulfate (DMS, 0.5% in ethanol) for 5 min and stopped by addition of 10% glycerol and β-mercaptoethanol. Samples were separated onto a 15% native polyacrylamide gels (acrylamide/bis solution 19:1). DNA bands were localized via autoradiography, excised and eluted overnight. The supernatants were ethanol-precipitated and treated with piperidine 1 M for 30 min at 90°C. Samples were dried and washed in a speed-vac, and resuspended in formamide gel loading buffer. Reaction products were analyzed on 20% denaturing polyacrylamide gels (acrylamide/bis solution 19:1) containing 8 M urea, visualized by phosphorimaging analysis on a Typhoon FLA 9000 (GE Healthcare Europe, Milan, Italy), and quantified by ImageQuant TL software (GE Healthcare Europe, Milan, Italy).

### NMR analysis

Oligonucleotides for NMR experiments were synthesized on a K&A Laborgeraete GbR DNA/RNA Synthesizer H-8 using standard phosphoramidite chemistry in DMT-on mode, deprotected by Glen-Pak cartridges and desalted on a Sephadex G25 column. NMR experiments were performed on the 600 MHz Agilent-Varian DD2 spectrometer with HCN cold probe and 600 MHz Bruker Avance NEO spectrometer with cryo probe. NMR spectra were acquired in 20 mM potassium phosphate buffer, pH 7.4, 100 mM K^+^ or TE buffer, pH 8.0, 100 mM K^+^ and 25°C. Oligonucleotide concentration was between 0.5 and 0.8 mM. Spectra were processed and visualized with Mnova (Mestrelab Research), NMRPipe and NMRFAM-SPARKY programs (2D).

### Taq polymerase stop assay

The DNA primer (Supplementary Table S1) was 5′-end labeled with [γ-^32^P]ATP using T4 polynucleotide kinase (Thermo Scientific, Milan, Italy) at 37°C for 30 min and then purified with Illustra MicroSpin G-25 columns (GE Healthcare, Milan, Italy). The labeled primer (final concentration 72 nM) was annealed to the template (final concentration 36 nM) (Supplementary Table S1) in lithium cacodylate buffer (10 mM, pH 7.4) in the presence or absence of KCl (20 mM) by heating at 95°C for 5 min and gradually cooling to room temperature to allow both primer annealing and G4 folding, and incubated overnight. Where indicated, the G4-ligands c-exNDI and Quarfloxin were added at the concentrations 12.5, 50 and 200 nM with the G4 template, and 200 nM only with the negative control template. The primer was subsequently extended on the template strand by adding 2 U/reaction of AmpliTaq Gold DNA polymerase (Applied Biosystem, Carlsbad, CA, USA) at 56°C for 30 min. Reactions were stopped by ethanol precipitation and primer extension products were separated on a 16% denaturing gel, and finally visualized by phosphorimaging (Typhoon FLA 9000). Markers were prepared based on the Maxam & Gilbert sequencing by PCR reaction with ^32^P-labeled primer. PCR products were treated with formic acid for 5 min at 25°C and then with piperidine for 30 min at 90°C.

### Four-tetrad pG4s prediction

The Quadparser G4 prediction tool (23) was employed to calculate pG4s in the complete human genome (hg19, GRCh37 https://www.gencodegenes.org/human/) and in the OQs sequences obtained by a modified G4-sequencing protocol developed by Chambers *et al.* (24). OQs genomic positions (bed file) were downloaded from GEO (accession GSE110582) (25), converted in the FASTA format by mean of bedtools (26) using hg19 as reference genome, and analysed with Quadparser for the prediction of pG4s. Five pG4s groups were analysed base on the categories described by Marsico *et al.* (25). The regular expressions used in Quadparser for each pG4s category are the following:

1. **G_3+_L_1–7_** = canonical pG4s, with at least three tetrads and loops of length up to seven nucleotides: ‘([gG]{3,}\w{1,7}){3,}[gG]{3,}’;
2. **G_3+_L_1–12_** = extended pG4s, with at least three tetrads and longer loops up to 12 nucleotides: ‘([gG]{3,}\w{1,12}){3,}[gG]{3,}’;
3. **G_4_L_1–7_** = canonical four-tetrad pG4s, with at least four tetrads and loops of length up to seven nucleotides: ‘([gG]{4}\w{1,7}){3,}[gG]{4}’;
4. **G_4_L_1__–__12_** = extended four-tetrad pG4s, with at least four tetrads and loops up to 12 nucleotides ‘([gG]{4}\w{1,12}){3,}[gG]{4}’;
5. **G_4_L_3_** = *MDM2*-like pG4s, with at least four tetrads and loops of three nucleotides ‘([gG]{4,}\w{3}){3,}[gG]{4}’.

## RESULTS

### G4 computational prediction in the *MDM2* P2 inducible promoter

The first intron of the *MDM2* human oncogene contains the inducible promoter P2, which allows faster processing of transcripts with respect to the constitutive promoter P1 and thus is the mainly responsible for MDM2 overexpression in different types of cancer (27). We observed that the P2 promoter sequence is extremely G-rich and thus we set out to investigate the possibility that it folded into G4s. We analyzed the 526 bp-long P2-containing intron of *MDM2* (GRCh38.p2, NC_000012.12) for the presence of putative G4 (pG4)-forming regions by QGRS Mapper (http://bioinformatics.ramapo.edu/QGRS/index.php). Eleven pG4s, 10 on the forward and 1 on the reverse strand, were identified (Table 1). These were named according to their position in the analyzed sequence. Eight pG4s were composed of GG-tracts, two of GGG- and one of GGGG-tracts; the likelihood to fold of each of these sequences was predicted by the G-score.

**Table 1.** pG4s identified in the P2-containing intron of *MDM2*

Name	Position^a^	Length	pG4 sequence^b^	G-score	Strand
pG4-21	21	28	GGTCACTTTTGGGTCTGGGCTCTGACGG	28	F
pG4-100	100	16	GGTTCGTGGCTGGGGG	27	F
pG4-119	119	25	**GGGGCGCGGGGCGCGGGGCATGGGG**	93	F
pG4-160	160	23	GGTTTTGTTGGACTGGGGCTAGG	28	F
pG4-195	195	14	GGGAGGAGGGCGGG	31	F
pG4-214	214	21	GGACGGCTCTCGCGGCGGTGG	26	F
pG4-236	236	11	GGTGGGGGTGG	31	F
pG4-262	262	34	GGGAGTTCAGGGTAAACGGTACGGGGGCCGGGGG	58	F
pG4-311	311	16	GGCGCGGGAGGTCCGG	30	F
pG4-377	377	26	GGGCGGGATTGGGCCGGTTCAGTGGG	53	F
pG4-434R	434	39	GGCTGCGAACGGGCAGAGGCTGGGAACCAGCGATAGAGG	27	R

^a^The indicated position corresponds to the distance from the first base of the *MDM2* intron 1 and the first base of the pG4 sequence.

^b^Gs predicted to be involved in G4 formation are underlined. The G4-forming sequence further investigated in the present work is highlighted in red.

**Figure 1.**
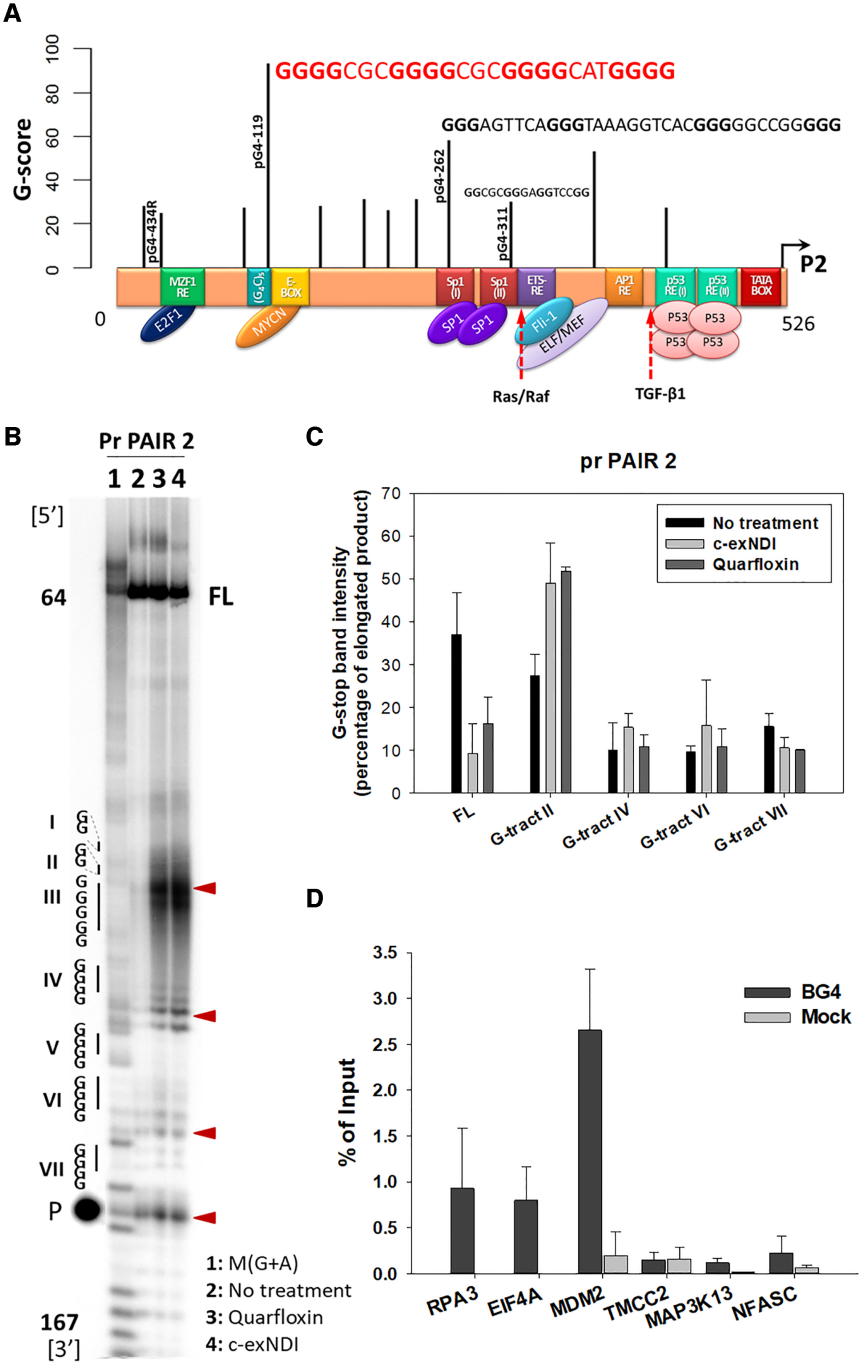
The MDM2 P2 promoter folds into multiple G4 sequences in vivo. (**A**) Position of the predicted G4s with respect to the regulatory regions contained in the P2. Numbers on the left and right of the promoter scheme correspond to the P2 promoter sequence bp count. QGRS score is reported on the y-axis. (**B**) Representative PCR stop assay of the G-rich MDM2 strand. Amplification products obtained with primers pair 2 are displayed. Relevant G-tracts present in the G-rich strand in the amplified region are reported on the left of the gel image according to the 5′–3′ orientation and identified by Roman numerals. The numbers 64 and 167 indicate the position of the amplified region with respect to the full-length MDM2 P2 sequence. The complete amplified sequence together with the stop sites are reported in Supplementary Table S2. Lane 1 is the amplified region treated according to the Maxam and Gilbert protocol to visualize Gs and As. Lanes 2, 3 and 4 are the PCR product amplified respectively in the absence of G4-ligand or in the presence of c-exNDI and Quarfloxin 200 nM. Red triangles indicate the PCR stops corresponding to G4 structures. ‘P’ indicates a lane loaded just with the PCR radiolabelled primer and ‘FL’ refers to the full length amplification product. (**C**) Quantification of lanes 2–4 of G4-compatible stops shown in panel B. Quantification of stop bands and standard errors corresponding to the indicated PCR stops and of the full-length amplification product (FL) are reported as mean of two independent experiments. (**D**) G4-ChIP followed by qPCR. Enrichment is reported as percentage of input. RPA3 and EIF4A were used as positive controls, while TMCC2, MAP3K13 and NFASC were negative controls that cannot fold into G4 structures.

PG4-119, the sequence that displayed the highest G-score, is located immediately adjacent to an E-box DNA motif (CACGTG) (Figure 1A). This type of element is known to act as enhancer to recruit transcription factors and thus to initiate gene transcription (28). PG4–119 is also embedded in the so called (nnGGGGC)_5_ repeated sequence, whose integrity is necessary for p53-independent activation of the P2 promoter (29). The second highest G-score sequence was pG4-262, which comprises one Sp1 binding site and the SNP285G>C locus (rs117039649), which forms a peculiar haplotype together with SNP309T>G (rs2279744). The C variant of SNP285C reduces the strength of Sp1 binding, counteracting the effect of SNP309G, which in contrast comprises a second Sp1 binding site leading to increased transcription of *MDM2* (30). This second Sp1 binding site corresponded to pG4-311, which also overlaps with an ETS response element. An efficient binding of ETS2 transcription factor to its binding site in the *MDM2* P2 promoter was shown to be necessary for p53-independent activation of P2 in breast cancer cells (29). PG4-434R, the only pG4 predicted on the reverse strand, was located in the region containing the MZF-1 response element, characterized by a G-rich consensus sequence (31). Therefore, at least 4 out of the 11 predicted pG4s (pG4-119, pG4-262, pG4-311 and pG4-434R) were located in or immediately adjacent to important transcription factor binding sites (Figure 1A). This observation suggests a potential role of these pG4s in the regulation of the *MDM2* P2 promoter.

### Investigation of *MDM2* G4 folding in the genomic context and in cells

To test if the *MDM2* G-rich region could actually fold into G4, we extracted the genomic DNA from 93T449 cells, a cell line originated from a retroperitoneal WDLPS, characterized by amplification (>90 copies per genome) (32) of the genomic region containing the entire *MDM2* gene. The extracted double-stranded DNA was subjected to PCR stop assay to check if the G-rich template strand could induce G4-mediated stops during polymerase amplification. A standard PCR reaction was performed in the presence of one radiolabeled primer, allowing to visualize the polymerase stops with single base resolution by sequencing gel electrophoresis. Addition of two known G4-ligands, namely c-exNDI and Quarfloxin, was used to verify their capacity to further stabilize *MDM2* G4s. The first has shown antitumoral effects against prostate cancer and melanoma with a consistently higher activity on cancer with respect to normal cells (18,33), the second is a small molecule disrupting nucleolin/rDNA G4 complexes in the nucleolus, thereby impairing ribosome biogenesis and inducing apoptosis in cancer cells (34). Since no more than 200 bp per sample can be resolved with this approach (35), we used six primer pairs that amplified 100–180 bp-long overlapping sequences to cover the whole *MDM2* P2 promoter region. A total of 13 main polymerase stops were observed: four in the 64–167 bp, four in the 143–285 and five in the 208–351 bp amplicon (Figure 1B and C, Supplementary Figure S1A and B, Supplementary Table S1). All polymerase stops were close to G-tracts involved in pG4s. In the 64–167 bp amplicon, the first stop (Figure 1B, lane 2) was right before the first G-tract (VII) encountered by the polymerase and corresponding to pG4-119. Two other stops were visible at G-tracts IV and VI, suggesting the presence of several G4 species. In the same amplicon, a fourth stop was observed right upstream the first G-tract (III) of pG4-100. In the presence of the two tested G4 ligands G4 stabilization was visible as increment of stop band intensity corresponding to both pG4s, resulting in a reduction of the full-length amplicon intensity from ∼40% to ∼10% and ∼15% for c-exNDI and Quarfloxin, respectively (Figure 1C). In the 143–285 bp amplicon, the four stops were compatible with: (i) pG4-195, (ii) pG4-214 and a non-predicted G4 involving four G-runs in between the two G-tracts 236 and pG4-214. Confirming this result, in the 208–351 bp amplicon the five observed stops were compatible respectively with: (i) pG4-214, (ii) pG4-236 and (iii) pG4-311. The G4-ligand stabilizing effect is however negligible on these G4s (Supplementary Figure S1A and B). These data strongly suggest that at least six distinct and highly stable G4 structures compatible with QGRS algorithm prediction can fold in the *MDM2* P2 promoter and that two of them can be further stabilized by the interaction with G4-ligands. Other pG4s were not detected by the described assay (Supplementary Figure S1), but their formation at lower physiological temperature cannot be excluded.

To extend the above observation to the cellular context, we performed G4-ChIP with the anti-G4 antibody BG4 (36), followed by qPCR. To check formation of G4s in the *MDM2* P2 promoter, we employed a primer pair spanning a 157 bp-long region centered in the pG4-densest portion. This region comprises: pG4-214, pG4-236, pG4-262, pG4-311 and pG4-377. High enrichment of the *MDM2* sequence was found (Figure 1D), confirming formation of G4s in this region in cells. To a lower extent, enrichment was also observed in *RPA3* and *EIF4A* promoters, used as positive controls, since they were already reported to fold into G4s and be efficiently recognized by BG4 (36). In addition these genes likely maintain similar expression in different cell lines (37,38). In contrast, the negative controls *TMCC2*, *MAP3K13* and *NFASC*, which do not contain any putative G4 sequence, were not enriched (Figure 1D). This result indicates that the *MDM2* P2 genomic region actually folds into G4 *in vivo*.

### *MDM2* G4 folding *in vitro*

G4-ChIP supplied *in vivo* evidence for G4 formation, but it could not provide sufficient resolution at the individual G4 level. To prove G4 formation *in vitro*, we selected sequence pG4-119 because (i) it folds within the genomic DNA of 93T449 cells (Figure 1B); (ii) it is embedded within key transcription regulatory regions (Figure 1A); (iii) it has the highest G-score, which indicates the putative highest stability (Table 1); (iv) it is composed of G-tracts of four/five Gs. Such a G-tract pattern in the human genome is much less frequent than tracts of three Gs and can theoretically fold into a four-tetrad G4. We analyzed two sequences: one comprising the five G-tracts (*Mdm2-G4*), and a second one that also included the 5′ flanking sequence that contains additional Gs (*Mdm2-G4-L*, Supplementary Table S2).

CD spectroscopy coupled to thermal unfolding in Li^+^ Cacodylate buffer was applied to analyze *Mdm2-G4-L* in the presence of 0, 20 and 100 mM K^+^, a monovalent cation that stabilizes G4s. *Mdm2-G4* was analyzed only at the physiological K^+^ concentration of 100 mM for comparison. *Mdm2-G4-L* CD analysis indicated G4-folding with increased stability at increasing K^+^ concentration (Table 2). In the absence of K^+^, *Mdm2-G4-L* adopted a hybrid conformation, while the antiparallel conformation prevailed upon addition of K^+^ (Supplementary Figure S2A–E). To exclude that the G4 structure observed in the absence of K^+^ was induced by the Li^+^ ions present in solution, CD analysis in the presence and absence of 100 mM K^+^ was also performed in different non-reactive buffers: MES, HEPES and Tris (Supplementary Figure S3, Supplementary Table S3). The same conformational difference observed in Li^+^ Cacodylate was also observed in the presence of the other buffers, with the highest K^+^ stabilizing effect observed in Li^+^ Cacodylate and HEPES (Supplementary Table S3). Since in the presence of Li^+^ Cacodylate *Mdm2* antiparallel conformation was most defined, this buffer was used in all the further analyses. *Mdm2-G4-L* and *Mdm2-G4* displayed almost superimposable CD spectra, signatures of antiparallel G4 (Supplementary Figure S2A), but the higher thermal stability of *Mdm2-G4-L* suggests that Gs in the 5′ flanking region also contribute to the overall G4 stability.

**Table 2.** CD Melting temperatures of *Mdm2-G4* and *Mdm2-G4-L* G4 oligonucleotides

Oligo	K^+^	Drug	*T*_m_^a^	Δ*T*_m_	Conformation
*Mdm2-G4*	100 mM	/	86.3 ± 0.8	/	Antiparallel
*Mdm2-G4-L*	100 mM	/	>90°C	/	Antiparallel
*Mdm2-G4-L*	20 mM	/	71.4 ± 2.3°C	/	Antiparallel
*Mdm2-G4-L*	0 mM	/	29.8 ± 1.3°C, 55.1 ± 0.6°C	/	Mixed/hybrid
*Mdm2-G4-L*	20 mM	c-exNDI	83.5 ± 1.4°C	14.8 ± 3.7°C	Antiparallel
*Mdm2-G4-L*	20 mM	Quarfloxin	>90°C	>18.6°C	Antiparallel

^a^The reported *T*_m_ values were calculated at the positive peak (∼291–295 nm) and represent the mean of two independent replicates. For the 0 mM K^+^ condition, the two indicated *T*_m_ values were calculated at the two positive peaks 260 and 283 nm, respectively.

### Identification of nuclear proteins specifically interacting with MDM2*-*G4

To assess if proteins interacting with G4s forming in sequence pG4-119 (from now on called MDM2-G4) existed, we performed a protein pull-down assay in WDLPS cell extracts, followed by LC–MS identification. As bait we used the five G-tracts *Mdm2-G4* sequence (*Mdm2-G4-biot*). Several controls were used to exclude non-specifically binding proteins: a single stranded non-G4 G-rich (*G-ss-biot*), a single stranded C-rich (*C-ss-biot*) sequence (Supplementary Table S2) and the pull-down beads lacking any oligonucleotides. Mascot scoring was employed to assign protein-matching confidence: proteins that were identified with a significant score (>20) were considered only if their score on the target was at least double than that on control sequences (Table 3). Among the pulled-down proteins displaying selectivity for MDM2-G4, we detected a distinct enrichment in helicase and unwinding proteins, with prevalence of the hnRNP family of ribonucleoproteins (Table 3). Besides hnRNPs, other proteins with secondary structure unwinding activity were identified: the protein dimer Ku70–Ku80, which is part of the RNA helicase II complex (39), and the RNA helicase DDX3X (40). We also identified a protein with G4 stabilizing effect, EWS that is involved in various cellular processes, among which transcriptional regulation (41). The abundance of WDLPS expressed proteins that recognize MDM2*-*G4 and possess secondary structure-resolving activity further supports the presence of G4s in the *MDM2* P2 promoter *in vivo*. These data also highlight the importance and necessity for the cell to unfold these G4s.

**Table 3.** LC–MS identification of MDM2*-*G4-specific binding proteins

Name	Function	Score^a^	Also found on	Reported activity
hnRNP A3	Trafficking of RNA	125	*C-ss* with similar score	Unwinding (72)
ACBL	involved in various types of cell motility	123	/	/
XRCC6	ss DNA-dependent ATP-dependent helicase	89	*G-ss*	Helicase (39,73)
XRCC5	ss DNA-dependent ATP-dependent helicase	57	*G-ss*	Helicase (39,73)
hnRNP F	Binds G-rich sequences in pre-mRNAs keeping it in an unfolded state	57	/	Unwinding (74)
DDX3X	Multifunctional ATP-dependent RNA helicase	29	/	Helicase (40)
EWS	transcriptional regulator	28	/	Stabilizing on G4s (57)
hnRNP Q	implicated in mRNA processing mechanisms	24	*G-ss* / *C-ss*	Forming a complex with DHX9 Helicase (75)
hnRNP H3	early heat shock-induced splicing arrest	22	/	Unwinding (76)

^a^Reported are the proteins that specifically bind MDM2-G4 and that were positively identified based on Mascot score: this represents the probability that protein identification was not a random event; it depends on the proportion of assigned peptides and peptide ion masses corresponding to a given protein.

### Targeting G4s in the MDM2 P2 promoter with a G4-ligand

Stabilization of G4s in oncogene promoters has been associated to transcription inhibition (33,42). We therefore envisaged that targeting the G4s in the *MDM2* P2 region with G4 stabilizing ligands would impair activation of P2-mediated transcription and counteract the unwinding proteins’ activity, thus restoring physiological levels of the MDM2 protein. To verify this hypothesis, we tested the possibility that MDM2-G4 interacted with the known G4-ligands c-exNDI and Quarfloxin. The compounds effect on *Mdm2-G4-L* thermal stability was tested in the presence of 20 mM K^+^, since G4 stability at higher K^+^ concentrations was already above 90°C: stabilization by about 15 and >21°C was observed in these conditions by addition of c-exNDI and Quarfloxin, respectively (Table 2 and Supplementary Figure S2F and G).

**Figure 2.**
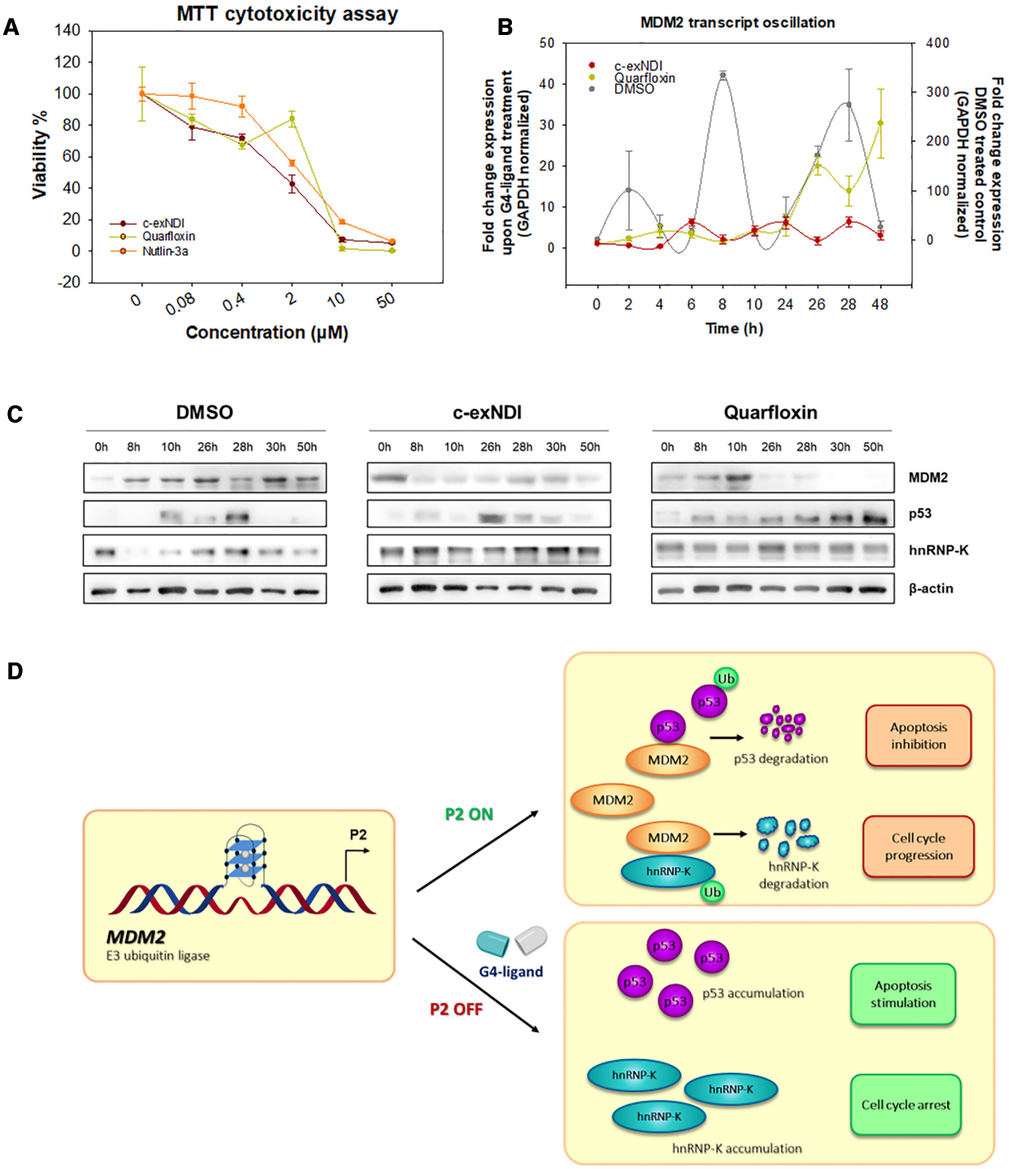
G4-ligands impairs MDM2 transcription and restores the integrity of MDM2 target proteins in 93T449 cells. (**A**) MTT cytotoxicity assay upon 48 h treatment in the presence of c-exNDI, Quarfloxin and Nutlin-3a. Compounds were tested in the 0.08–50 μM concentration range. (**B**) RT-PCR showing the fold change expression of *MDM2* mRNA over time (0, 2, 4, 6, 8, 10, 24, 26, 28, 48 h) upon treatment with the two G4-ligands c-exNDI and Quarfloxin (55 nM) or DMSO treated control. Fold change expression values were internally normalized over GAPDH housekeeping mRNA and reported using the sample collected at 0 h from treatment as reference). The reported values represent the mean of three independent replicates with standard errors. Y-axis ranges are different for the G4-ligand treated samples (left axis) and DMSO control (right axis). (**C**) Western Blot analysis on cellular lysates to evaluate c-exNDI and Quarfloxin (55 nM) effect over time (0, 8, 10, 12, 26, 28, 30, 50 h post-treatment) on MDM2 protein and its two targets p53 and hnRNP-K. DMSO treated lysate were evaluated as control sample. β-actin is shown as housekeeping gene. (**D**) Scheme of MDM2 pathways relative to its ubiquitin ligase activity on p53 and hnRNP-K, consequently inducing apoptotic arrest and stimulation of cell cycle progression (upper panel). Scheme of the proposed effect of G4-ligands on MDM2 P2-mediated transcription: the compound impairs *Mdm2* transcription by binding to *Mdm2* G4 (lower panel).

We next tested the cytotoxic effect of c-exNDI and Quarfloxin against 93T449 cells (32). Nutlin-3a, a potent MDM2 antagonist that inhibits MDM2-p53 interaction (20), was used for comparison. Interestingly, G4-ligands displayed CC_50_, i.e. the compound's concentration required to reduce cell viability by 50%, at least 2-fold higher (c-exNDI: 1.30 ± 0.27 μM; Quarfloxin: 1.02 ± 0.11 μM) than that of Nutlin-3a (2.63 ± 0.13 μM) (Figure 2A). The described *in vitro* data combined with the high cytotoxicity observed in WDLPS cells are good assumptions to support interference with the p53-MDM2 pathway by G4-ligand binding to *MDM2* P2 G4s (Figure 2D). All three compounds displayed higher cytotoxicity on a control normal cell line (HNLF) (CC_50_ values are 0.004 ± 0.001 μM for c-exNDI, 0.503 ± 0.047 μM for Quarfloxin and 0.722 ± 0.067 μM for Nutlin-3a) (Supplementary Figure S4), which is not surprising considering the inherent higher frailty of primary cells versus tumour cells in culture. The high cytotoxicity of c-exNDI in this cell line also implies that its potent binding to G4 structures is likely not limited to the MDM2 P2 G4.

### Exploiting G4-ligands to counteract WDLPS via the p53-MDM2 pathway

To assess if the G4-ligands c-exNDI and Quarfloxin affected MDM2 expression, we measured *MDM2* mRNA and protein levels upon treatment with the compounds. The G4-ligands were administered at subcytotoxic concentration (55 nM) to avoid accumulation of downstream effects, thus to allow assessment of the compound's direct targets. The MDM2 protein is known to be transcribed and expressed in a strictly regulated oscillatory wave that depends in turn on p53 expression (43–45). This allows for the correct accumulation or degradation of p53 and other MDM2 target proteins at the correct cell cycle phase or in the presence of threats to cell integrity. Cell survival is therefore strongly dependent on the tight modulation of MDM2-p53 levels. Therefore, for a correct evaluation of G4-ligand effect on MDM2 expression, its mRNA level was evaluated by RT-PCR at several time points (0, 2, 4, 6, 8, 10, 24, 26, 28, 48 h) after G4-ligand in DMSO or DMSO treatment alone, used as negative control (Figure 2B). A strong alteration of mRNA global accumulation and oscillatory phases were observed upon G4-ligand treatment. In particular, the transcribed mRNA amount was highly reduced, and despite an oscillatory pattern was still visible, the first MDM2 transcription peak wave was reached with a visible delay with respect to the control. The downstream effect of such alteration in MDM2 transcription was evaluated by measuring MDM2 protein levels, especially at long times post-treatment (0, 8, 10, 26, 28, 30, 50 h). As controls, we included two proteins that are involved in MDM2-regulated pathways (46). In particular, p53, the expression of which is downregulated by MDM2 E3 ubiquitin ligase activity in a regulatory feedback loop, and hnRNP-K, one of the proteins that are marked by MDM2 for proteasomal degradation and an important p53 co-factor for induction of cell-cycle arrest (47) (see scheme in Figure 2D). In general, the alteration in MDM2 protein oscillatory accumulation reflected the pattern observed by RT-PCR. Moreover, we observed a prevalent depletion of MDM2 protein paralleled by accumulation of both p53 and hnRNP-K (Figure 2C). Specifically, p53 protein started to accumulate early after treatment with both G4-ligands and it was kept at a higher level especially at long times (30–50 h) with respect to the DMSO-treated control that showed undetectable levels in these conditions. Accumulation of hnRNP-K protein was also observed upon treatment with both G4-ligands, with its accumulation in the cell being faster and kept to a more homogeneous level along time. MDM2 altered expression, which induces accumulation of its direct targets, such as p53 and hnRNP-K, points to the potential stimulation of cell defence mechanism which would result in cell death or cell cycle arrest.

**Figure 3.**
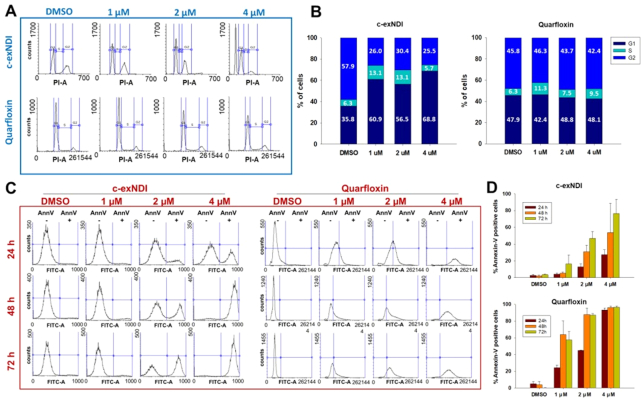
FACS evaluation of cell cycle and apoptosis after c-exNDI treatment. (**A**) Effect on cell cycle progression evaluated at 24 h upon treatment with 1, 2 and 4 μM of c-exNDI and Quarfloxin. The cell cycle profile of a representative replicate is shown. (**B**) Quantification of cell cycle phases shown in panel A. The mean of three independent replicates is shown. Standard error was lower than 10%. (**C**) Detection of apoptosis upon treatment with 1, 2 and 4 μM c-exNDI and Quarfloxin for 24, 48 and 72 h and annexin V staining. Profiles for one representative replicate is shown. (**D**) Quantification of annexin V positive cells shown in panel B. Values corresponding to the mean of two independent replicates with standard error are reported.

To establish if these latter downstream effects occurred, we performed FACS analysis upon treatment with c-exNDI and Quarfloxin. Based on the measured CC_50_, the compounds were administered in a range of concentrations (1–4 μM) sufficient to yield proliferative alterations according to the MTT assay. Indeed, upon c-exNDI treatment we observed dose-dependent cell growth arrest in G1 phase and sub-G1 phase, which is indicative of apoptosis (Figure 3A and B). The degree of apoptosis was further evaluated by annexin V staining: we obtained a dose- and time-dependent increase in the apoptotic cell fraction, which reached values higher than 80% with respect to the total cell fraction after 72 h of treatment at 4 μM (Figure 3C and D). Quarfloxin treatment, showed no obvious alteration of the cell cycle, but consistent induction of apoptosis (Figure 3A–D). The stable levels of hnRNP-K protein upon Quarfloxin treatment previously observed may account for the absence of major cell cycle alterations. Moreover, the more linear accumulation of p53 upon Quarfloxin treatment (Figure 2C and D) can be explained by the different principal mechanisms of action of Quarfloxin and c-exNDI. Quarfloxin binding to rRNA G4 relocalized nucleolin in the cytoplasm where it prevents MDM2-p53 complex formation by binding to both MDM2 and p53 (48). Thus, p53 stabilization most likely results from of a double action of the compound: MDM2 suppression at both transcriptional and protein activity level.

These findings indicate that G4-ligands that can bind and stabilize G4s in the *MDM2* P2 region inhibit *MDM2* transcription, in turn leading to the reactivation of the pathways involving MDM2 target substrates. In particular, the reduced degradation of p53 and hnRNP-K results in the induction of both cell cycle arrest and apoptosis in WDLPS cell.

### Biophysical characterization of MDM2-G4

We reasoned that the possibility to target specific G4s in the *MDM2* P2 promoter among those that can form in the cell would likely allow enhanced effects. We estimated that the MDM2-G4 sequence, i.e. pG-119, possessed the structural features that could be later exploited for the design of selective G4-ligands and ultimately selective targeting. In fact, besides being in a key genome position, it could possibly display the uncommon antiparallel conformation and folding into four-tetrad G4. The latter is also a quite rare structure, as we proved by applying Quadparser (23) both on the complete human genome (GRCh37) and on the observed quadruplexes (OQs) detected by a modified G4-sequencing protocol (24). We calculated the abundance of canonical and extended four-tetrad pG4s (G_4_L_1–7_ and G_4_L_1–12_, respectively) (Supplementary Table S4 and Supplementary Figure S5): these were found to be only a small fraction, i.e. 8–11% and 9–19%, respectively, of all the pG4s and OQs. Having *MDM2* a very regular G4 pattern, with three nt-long loops, we also calculated the abundance of *MDM2*-like pG4s (G_4_L_3_): <0.6% of pG4s/OQs were found with such features.

**Figure 4.**
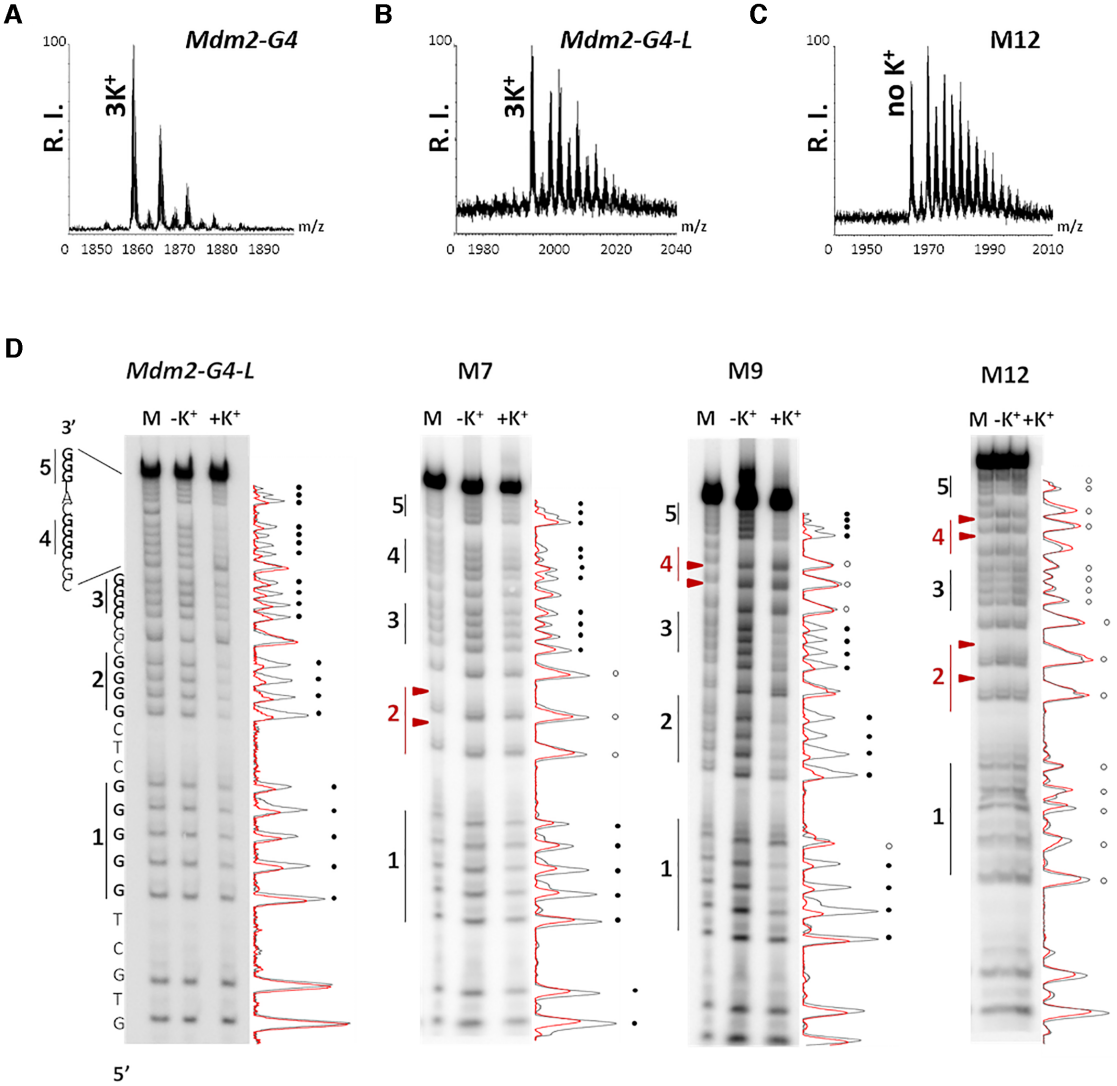
Biophysical characterization of MDM2-G4. LC-MS analysis of (**A**) *Mdm2-G4*, (**B**) *Mdm2-G4-L* and (**C**) the most destabilized mutant M12. The reported spectra correspond to the most defined charge state, i.e. −6 for *Mdm2-G4* and −7 for the other oligonucleotides. (**D**) DMS footprinting of *Mdm2-G4-L* and M7, M9 and M12 mutants. For each oligonucleotide, Lane 1 is the G+A marker, lanes 2 and 3 correspond to the DMS reaction in the absence and presence of K^+^, respectively. Densitogram-based band quantification is reported on the left of each gel (the black and red lines correspond to samples folded in the absence and presence of K^+^, respectively). Black circles indicate protected Gs. White circles indicate unprotected or partially protected Gs. Red triangles indicate mutated Gs.

The presence of ≥4 Gs in G-tracts does not necessarily imply formation of a four-quartet G4, as a variety of G4s formed by less quartets can be present, similarly to what observed in the human telomeric sequence (49). Since each G4 quartet pair is stabilized by one atom of monovalent cation, mass spectrometry (MS) can diagnose the number of stacked quartets (22). As shown in Figure 4A and B and Supplementary Table S5, both *Mdm2-G4* and *Mdm2-G4-L* coordinated three K^+^ ions, corroborating their folding into four-quartet G4s.

Since only four G-tracts are necessary and sufficient to form a monomolecular G4, the *Mdm2-G4-L* sequence (derived from the (nnGGGGC)_5_ region) must have a prevalent conformation that excludes one G-tract, or alternatively, more than one G4 conformations may be present in equilibrium. To obtain hints about the Gs involved in MDM2-G4, we performed DMS-footprinting on *Mdm2-G4-L* and 12 mutants (M1–12) in which one or more Gs were substituted with thymines (Supplementary Table S2). Since DMS-footprinting on *Mdm2-G4* did not allow to visualize all nucleotides involved in G4 formation (Supplementary Figure S6B), the *Mdm2-G4-L* extended sequence was preferred to correctly evaluate G4-involved Gs. DMS-footprinting of *Mdm2-G4*-*L* revealed that all five G-tracts are involved in G4 formation, with a more intense protection of the second and fourth G-tracts, suggesting equilibrium between two or more conformations (Figure 4D). Mutants of the central G-tract 3, namely M1–3 and M8, forced formation of a long central loop and therefore strongly reduced *Mdm2-G4-L* thermal stability measured by CD thermal unfolding (Δ*T*_m_ > 10°C) (Supplementary Figures S6A and S7, Supplementary Table S6) (50). In contrast, mutations of the external G-tracts 1 and 5 (M4–6, M10), impacted the overall G4 stability to a lower extent, shifting the equilibrium to G4s involving the remaining G-tracts. Importantly, single base mutations in G-tracts 1 and 3, and double mutations in G-tract 5 were sufficient to completely exclude the mutated tracts from the resulting G4 structure (Supplementary Figure S6A), further indicating that the four-tetrad G4 is highly favored over the three-quartets species. On the contrary, mutations in G-tracts 2 and 4 (M7 and M9, respectively) strongly altered the original G4 conformation, which shifted from antiparallel to hybrid, albeit preserving high thermal stability (Supplementary Figures S6A and S7, Supplementary Table S6). G-tract 2 double mutant (M7) was still able to fold into G4, albeit with a different topology (Supplementary Figures S6A and S7). When G-tract 4 was mutated (M9), the remaining G-tracts were involved in G4 formation to a higher extent. These data suggest that G-tracts 2 and 4 are involved in the prevalent MDM2-G4-folded structure, while tracts 1, 3 and 5 are alternatively engaged in dynamic equilibria. Indeed, simultaneous mutation of G-tracts 2 and 4 (M12) yielded non-G4 DMS-profile and CD features (Figure 4D, Supplementary Figures S6A and S7, Supplementary Table S6). MS further supported the above observations, as M12 did not coordinate any K^+^ ions (Figure 4C). Finally, mutation of the 5′-flanking bases (M11) did not alter the G4-like DMS profile, suggesting that their stabilizing effect was not due to Hoogsteen hydrogen bonds involved in G4 folding (Supplementary Figures S6A and S7, Supplementary Table S6).

**Figure 5.**
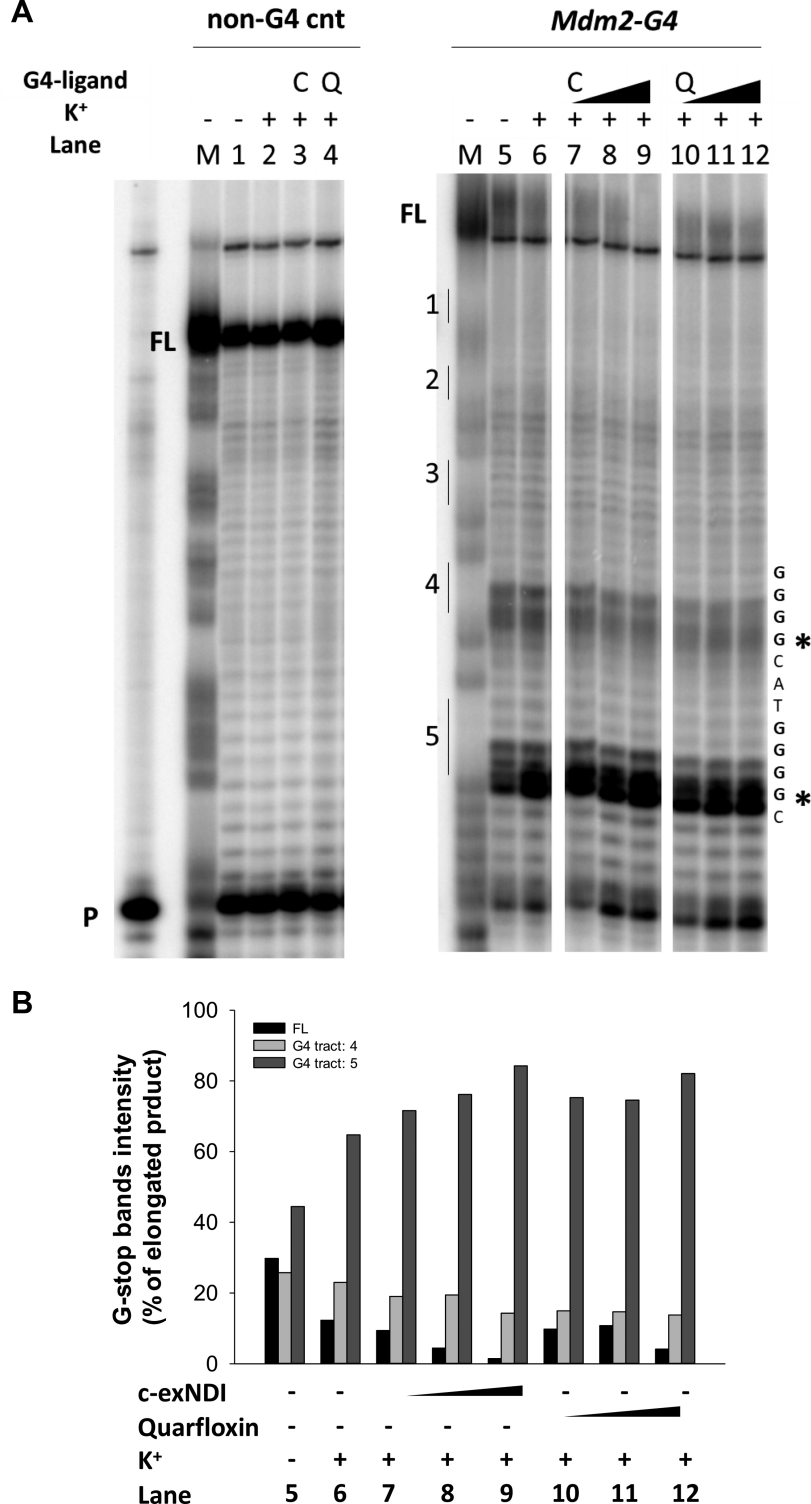
MDM2 G4 folds into multiple species. (**A**) Image of a typical Taq polymerase stop assay. *Mdm2-G4* template was amplified by Taq polymerase at 56°C in the absence (lane 5) and presence (lane 6) of 20 mM K^+^, 12.5, 50 and 200 nM c-exNDI (lanes 7–9) and Quarfloxin (lanes 10–12). A template (non-G4 cnt) made of a scrambled sequence unable to fold into G4 was also used as negative control (lanes 1–4). Lane P: unreacted labeled primer. Lane M: ladder of markers obtained by the Maxam and Gilbert sequencing carried out on the amplified strand complementary to the template strand. Vertical bars indicate position of the G-tracts, while asterisks indicate G4-specific Taq polymerase stop sites. (**B**) Quantification of lanes 5–12 shown in panel A from two independent experiments. Quantification of stop bands corresponding to *Mdm2-G4* and of the full-length amplification product (FL) is shown.

The G-tracts involved in MDM2-G4 formation were further investigated by Taq polymerase stop assay performed on an extended *Mdm2-G4* oligonucleotide (Supplementary Table S1). Two stops corresponding to G-tracts 5 and 4 of *Mdm2-G4*, i.e. the first and second G-tracts encountered by the polymerase, were visible both in the presence and absence of K^+^. Upon addition of 20 mM K^+^, intensity of the bands corresponding to G-tract 5 increased, resulting in a consistent reduction of the full-length amplicon (Figure 5A, lanes 5 and 6, and B). This result corroborates the fact that MDM2-G4 folds even in the absence of K^+^, and that K^+^ further stabilize them. The presence of multiple dynamic MDM2-G4 species, including the one that exclude G-tract 5 from the folding, was confirmed by the presence of the two stops. In contrast, no polymerase stops were visible on a DNA template unable to fold into G4 (Figure 5A, lanes 1–2), indicating that the observed polymerase inhibition was G4-dependent. The addition of the two G4-ligands c-exNDI and Quarfloxin was able to further stabilize MDM2-G4, as demonstrated by the dose-dependent increase of G4 stop bands intensity and reduction of the full-length amplicon (Figure 5A, lanes 7–12), with an effect which is mainly visible on G-tract 5, the first encountered by the polymerase (Figure 5B).

1D NMR analysis was initially performed on the *Mdm2-G4* sequence in the presence of 100 mM K^+^ (Supplementary Figure S8A). Number of proton signals in the imino region corresponding to Hoogsteen hydrogen-bonded protons in G-quartets denoted formation of several structures as suggested by DMS and Taq polymerase stop assay data. To reduce the number of possible different conformations, we split *Mdm2-G4* into two oligonucleotides, comprising tracts 1–4 (*Mdm2-G4,1-4*) and 2–5 (*Mdm2-G4,1-5*), respectively (Supplementary Table S2). CD spectra of the two oligonucleotides recorded in the presence of 100 mM K^+^ confirmed the conservation of the highly stable (*T*_m_ > 90°C) antiparallel G4 adopted by the full-length *Mdm2-G4* (Supplementary Figure S9). Both oligonucleotides exhibit formation of more than one structure in the presence of 100 mM K+. However, for both oligonucleotides it was possible to observe 16 partially resolved proton signals with high intensity in the region from δ 11–12 ppm (Supplementary Figure S8B and C), confirming the prevalent four-quartet G4 conformation. Detailed analysis of 2D NOESY spectra of *Mdm2-G4,1-4* showed two groups of four partially overlapped G residues in *syn* conformation (Supplementary Figure S8D and E) corroborated by their strong H1′-H8 and weaker H2′/H2″-H8 cross-peaks of the same G residues in *syn* conformation. Noteworthy, H2′/H2″ resonances are downfield shifted with respect to standard chemical shifts. Their resolved character allows assignment of inter-residual H2′/H2″-H8 cross-peaks and sequential connectivities of residues in *syn* conformation to residues in *anti* conformation (Supplementary Figure S8E). Eight *syn-anti* steps are a signature of an antiparallel topology with four G-quartet planes, which is in agreement with CD spectra (Supplementary Figure S9). Interestingly, residues in *syn* conformation in the A and B groups have very similar chemical shifts indicating repetitiveness in the G4 structure, which in turn results in very similar chemical environment of respective nuclei. Repetitive dinucleotide steps within the G4 structure can be expected for four quartet structure due to alternating *syn–anti–syn–anti* pattern along the strands. In support, similar chemical shift distribution of *syn* G residues was observed for other four quartet antiparallel structures (51–54). A similar grouping of residues in *syn* conformation was also observed for *Mdm2-G4 and Mdm2-G4,2-5* albeit with more severe spectral overlap (Supplementary Figure S8F). To note that in the NMR data analysis it has been assumed that the predominant structure for all three oligonucleotides is monomeric, with mass spectroscopy confirming such assumption for *Mdm2-G4* (Figure 4A). In the case of dimeric or higher-order structures hypothetical antiparallel topology could not be based on the eight observed *syn-anti* steps.

**Figure 6.**
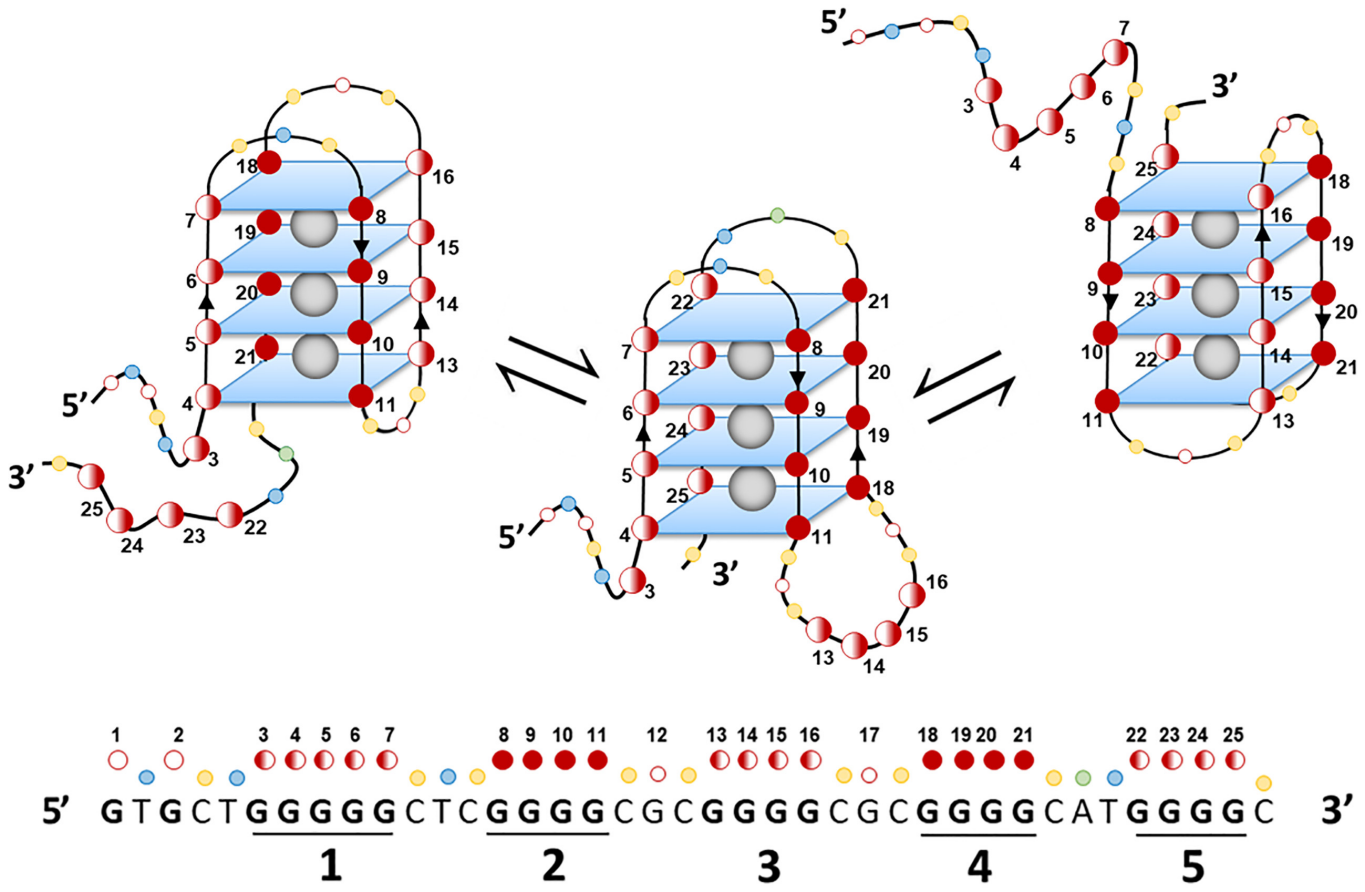
Model of MDM2 G4 four-tetrad antiparallel structure. Scheme of the three main MDM2-G4 forms deduced from DMS, CD, LC-MS and NMR analysis. Fully filled red circles indicate the Gs responsible for the prevalent specie, while half-filled circles are the ones present in different equilibria.

The integration of CD, DMS-footprinting, LC–MS, NMR and Taq polymerase stop analysis led us to generate the model schematized in Figure 6. The *MDM2* G-rich region adopts a unique antiparallel four G-quartet G4 structure, with G-tracts 2 and 4 that drive folding into the antiparallel conformation. G-tracts 2 and 4 are alternatively engaged in dynamic equilibrium with the remaining three G-tracts (G-1, G-3, G-5). The fact that at least four Gs in internal G-tracts must be mutated to disrupt MDM2-G4 folding highlights the high degree of conservation of the G4 folding and the importance of its integrity. All these are highly valuable prerequisites for the development of *MDM2* G4 selective targeting.

## DISCUSSION

Inguinal and retroperitoneal LPSs are high risk tumours due to their difficult treatment with standard approaches and tendency to relapse and progress (4–6). *MDM2* amplification and overexpression is the prevalent alteration responsible for LPS aggressiveness. Since the tumour suppressor p53 often remains wild-type in MDM2-overexpressing cancers, targeting MDM2 to restore p53 activity is a convincing antitumoral strategy. Several drugs that target the MDM2–p53 protein interaction have been tested; some of them reached clinical trials (e.g. HDM201 combined with LEE011, RG7112 and RG7388), but they were retired due to different reasons, comprising limited response rates and haematological toxicity (8,9,55). Thus, new therapeutic approaches and drugs are needed to successfully fight LPS.

So far no attempt has been made to specifically target *MDM2* at the transcriptional level, despite the fact that transcriptional targeting of the *MDM2* homolog *MDMX* has shown promising results (56). The high G-richness of the *MDM2* inducible promoter could conceivably provide targetable G4s to abrogate *MDM2* transcription, and thus reconstitute p53-mediated stimulation of cancer cell apoptosis.

Computational prediction revealed eleven pG4s within or in close proximity to key regulatory regions of the *MDM2* P2 promoter (12). PCR stop assay on the whole P2 promoter and G4 ChIP-qPCR on the genome of the WDLPS cell line 93T449 confirmed the *in vivo* existence of G4s in the *MDM2* promoter in WDLPS cells. The presence and importance of these in cells were further supported by identification of nine proteins, expressed in WDLPS cells, that selectively recognize MDM2-G4 with respect to unstructured G-rich and random ssDNA sequences. Most of the identified proteins have been previously reported to interact with G4s: seven of them are either helicases or can unwind complex nucleic acids structures (Table 2), while one is a G4-stabilizing protein (57). These data highlight the strict cellular requirement to unfold the G4s in the *MDM2* P2 promoter in the WDLPS environment and strongly support the hypothesis that *MDM2* G4s are key to the correct processing of the *MDM2* gene at the DNA level. The redundancy of modulatory factors with analogous function further emphasises the fundamental role played by MDM2 in the regulation of cell survival, so that if the main mechanism is damaged, alternative proteins can supply to the required function.

Targeting G4–protein complexes can be an effective therapeutic approach. For instance, the small molecule Quarfloxin was successfully employed to disrupts Nucleolin binding to rRNA G4s, resulting in anticancer effects (34). Counteracting the protein unwinding activity of G4s in *MDM2* P2 with G4-stabilizing ligands would likely represent a promising strategy to interfere with *MDM2* transcription. In addition to the previously mentioned G4-ligand Quarfloxin, c-exNDI (18,19) was also tested here as a proof-of principle *MDM2* inhibitor. The two compounds showed the ability to interact and stabilize MDM2-G4 and to exert twice as much cytotoxicity on WDLPS cells as Nutlin-3a, which is the most active enantiomer of the Nutlins’ family in the prevention of MDM2 interaction with p53 and other pro-apoptotic targets (58,59). Transcriptional downregulation of the *MDM2* homologous gene *MDMX* has been previously investigated (56). The small molecule NSC207895 was identified as promising *MDMX* inhibitor to restore p53 activity in cancer cells characterized by MDMX overexpression (56). Our approach, i.e. targeting *MDM2* P2 with G4-ligands, represents the first attempt to transcriptionally inhibit the MDM2 pathway. It is conceivable that G4s in *MDM2* P2 are major, but not unique targets for c-exNDI and Quarfloxin in WDLPS cells. In fact, WDLPS cells present a high copy number of the MDM2 gene (>90 copies) (32), but both c-exNDI and Quarfloxin are strong G4 ligand, reported to bind different G4 structures (19,33,60). Even so, G4-ligand treatment induced the alteration and general suppression of MDM2 both at the transcriptional and protein level and, being MDM2 a short-lived protein, the consequent downstream effects were already visible after 4/8 h of treatment. We observed accumulation of p53 and hnRNP-K proteins, both of which are direct targets of MDM2 for proteasomal degradation (7,47). Depending on the main mechanism of action of the tested G4-ligands, WDLPS cell G1 phase cycle arrest and apoptosis, which are known to be initiated by hnRNP-K and p53 respectively (61,62), corroborated both G4-ligand-mediated reactivation of the MDM2 target pathways and G4 ligand anticancer activity.

Obviously, the availability of a peculiar G4 structure would allow the development of more selective G4 ligands and thus the possibility to downregulate the MDM2 protein and restore its cellular targets, stimulating cell cycle arrest and apoptosis even more potently. In this direction, the pG4-119/MDM2-G4 sequence ((nnGGGGC)_5_) stood out for its folding into four-tetrad antiparallel G4s: a model of its 3D spatial arrangement into multiple four-tetrad species is reported in Figure 6. We have shown that four-tetrad G4s are a small fraction of the G4s that were calculated or observed in the human genome (24). In addition, pure antiparallel G4s are extremely rare: up to date, the *d*(GGGGCC)_4_ non-coding region from *C9orf72* (52) is the only well characterized human antiparallel G4 in physiological conditions. This observation, along with MDM2-G4 sequence closeness to an E-box enhancer that is recognized by the MYCN transcription factor (63) and its master role in P2-mediated transcription (29), supports the suitability of MDM2-G4 as exclusive target to allow for high therapeutic selectivity and potency.

The MDM2-G4 sequence is also characterized by the possibility to fold into alternative G4s (Figure 6). In particular, the *in vitro* characterization of the structure using different biophysical approaches suggested the presence of three main topological arrangements. Promoters where G-tracts contain different numbers of G bases and thus allow formation of multiple and dynamic G4 conformations have been reported both in humans and other organisms (64–66). Intriguingly, it is suggested that the equilibria of the folded state expose different recognition motifs to potential binding partners, thereby influencing G4 stability and possibly function (67). In addition, the fifth G-tract has been proposed as protection or spare motif to keep G4 folding in case of oxidative damage at G bases (68).

These observations coupled to MDM2*-*G4 structural data indicate high conservation of the overall G4 conformation in the *MDM2* P2 promoter and thus further point to a relevant biological role for the *MDM2* G4s.

The discovery of such extra stable G4s, which we showed to represent physical obstacles to polymerase processivity, may help shed light on the genetic origin of WDLPS. In fact, formation of non-canonical structures during DNA metabolism is a commonly observed cause of genome instability (69). *MDM2* amplification in WDLPS is associated to the formation of giant ring marker chromosomes containing multiple copies of the gene. Chimeric and fusion transcripts arising from such unstable regions are hypothesized to push the genomic amplification process during tumour progression (70). The deficiency of one or more *MDM2* G4 binding/unwinding proteins may cause replicative stress that worsens or is at the root of the pathological genome amplification, as recently proposed for ATRX-deficient malignant glioma (71).

In conclusion, our results indicate that (i) the P2 inducible promoter of the *MDM2* oncogene can fold into highly stable four-tetrad G4s; (ii) targeting *MDM2* G4s with G4-ligands represents a feasible strategy against WDLPS; (iii) the unique structural features of MDM2-G4 may allow the design of highly selective G4-ligands; (iv) the demonstrated G4 richness in the *MDM2* P2 promoter points to genetic instability and thus a mechanism for *MDM2* expansion and overexpression that leads to the tumoral phenotype. These data pave the way to a completely new approach to defeat LPS cells and thus cure WDLPS and in principle all tumours that overexpress MDM2.

## DATA AVAILABILITY

No dataset, software or tools have been deposited in online repositories. Additional material or information can be provided upon request by contacting the correspondig author.

## Supplementary Material

gkaa1273_Supplemental_File
