# Supplementary material for: The MDM2 inducible promoter folds into four-tetrad antiparallel G-quadruplexes targetable to fight malignant liposarcoma
Source: Nucleic Acids Res. 2021 Jan 7;49(2):847–63. doi: 10.1093/nar/gkaa1273 (PMC7826256; doi:10.1093/nar/gkaa1273)
Supplement: gkaa1273_Supplemental_File [file gkaa1273_supplemental_file.pdf]

Supplementary Information

The *MDM2* inducible promoter folds into four-tetrad antiparallel G-quadruplexes targetable to fight malignant liposarcoma

Sara Lago,[1] Matteo Nadai,[1] Emanuela Ruggiero,[1] Martina Tassinari,[1] Maja Marušič,[3] Beatrice Tosoni,[1] Ilaria Frasson,[1] Filippo M. Cernilogar,[2] Valentina Pirota,[4] Filippo Doria,[4] Janez Plavec,[3] Gunnar Schotta,[2] Sara N. Richter[1]*

[1]Department of Molecular Medicine, University of Padua, via A. Gabelli 63, 35121 Padua, Italy.

[2] Division of Molecular Biology, Biomedical Center, Faculty of Medicine, LMU Munich, Germany.

[3] Slovenian NMR center, National Institute of Chemistry, Hajdrihova, 19, Ljubljana SI-1000, Slovenia.

[4] Department of Chemistry, University of Pavia, V. le Taramelli 10, 27100, Pavia, Italy.

* To whom correspondence should be addressed. Tel: +39 0498272346; Email: sara.richter@unipd.it

Supplementary Information

Reported below are the Supplementary tables and figures containing: the sequence and G-tracts numbering of PCR Stop assay amplicons observed in Figure 1B of the main text; the name and sequence of the oligonucleotides and primers used in this study; PCR stop assay gels; Circular Dichroism (CD) unfolding spectra and melting temperatures (T_m); Dimethylsulphate footprinting experiments: MTT assay of HNLF and NMR spectra.

Supplementary Table 1. PCR stop assay on the complete *MDM2* P2 sequence. The six amplicons covering the *MDM2* P2 promoter are reported. The bp position of each amplicon with respect to the P2 promoter is indicated. Primer sequences are reported in blue, the pG4s predicted by QGRS are underlined and G-tracts are in bold. Red triangles indicate the position of the observed PCR stops (Figure 1B). The further analyzed MDM2-G4 sequence (see main text for details) is highlighted in red.

Pr PAIR	AMPLICON (bp)	SEQUENCE 5'-3' WITH STOPS	pG4s
1	-18 - 106	CCGGATGGTGAGGAGCAGGTACTGGCCCGGCAGCGAGCGGTCACTT TTGGGTCTGGGCTCTGACGGTGTCCCCTCTATCGCTGGTTCCCAGCCT CTGCCCGTTCGCAGCCTTTGTGCGGTTCG	pG4-21<br>pG4-434R
2	64 - 167	CTGGTTCCCAGCCTCTGCCCGTTCGCAGCCTTTGTGCGGTTCGTGGCT GGGGGCTCGGGGCGCGGGGCGCGGGGCATGGGGCACGTGGCTTT GCGGAGGTTTTG	pG4-100<br>pG4-119
3	143 - 285	GGCACGTGGCTTTGCGGAGGTTTTGTTGGACTGGGGCTAGGCAGTC GCCGCCAGGGAGGAGGGCGGGATTTCGGACGGCTCTCGCGGCGGT GGGGGTGGGGGTGGTTCGGAGGTCTCCGCGGGAGTTCAGGGTAAA GGTCACG	pG4-160<br>pG4-195<br>pG4-214<br>pG4-236
4	208 - 351	GGATTTCGGACGGCTCTCGCGGCGGTGGGGGTGGGGGTGGTTCGG AGGTCTCCGCGGGAGTTCAGGGTAAAGGTCACGGGGGCCGGGGGC TGCGGGGCCGCTTCGGCGCGGGAGGTCCGGATGATCGCAGGTGCCT GTCGGGTC	pG4-214<br>pG4-236<br>pG4-262<br>pG4-311
5	325 - 458	GGATGATCGCAGGTGCCTGTCGGGTCACTAGTGTGAACGCTGCGCGT AGTCTGGGCGGGATTGGGCCGGTTCAGTGGGCAGGTTGACTCAGCT TTTCCTCTTGAGCTGGTCAAGTTCAGACACGTTCCGAAAC	pG4-377
6	405 - 547	GGTTGACTCAGCTTTTCCTCTTGAGCTGGTCAAGTTCAGACACGTTCC GAAACTGCAGTAAAAGGAGTTAAGTCCTGACTTGTCTCCAGCTGGGG CTATTTAAACCATGCATTTTCCCAGCTGTGTTCAGTGGCGATTGGAG	/

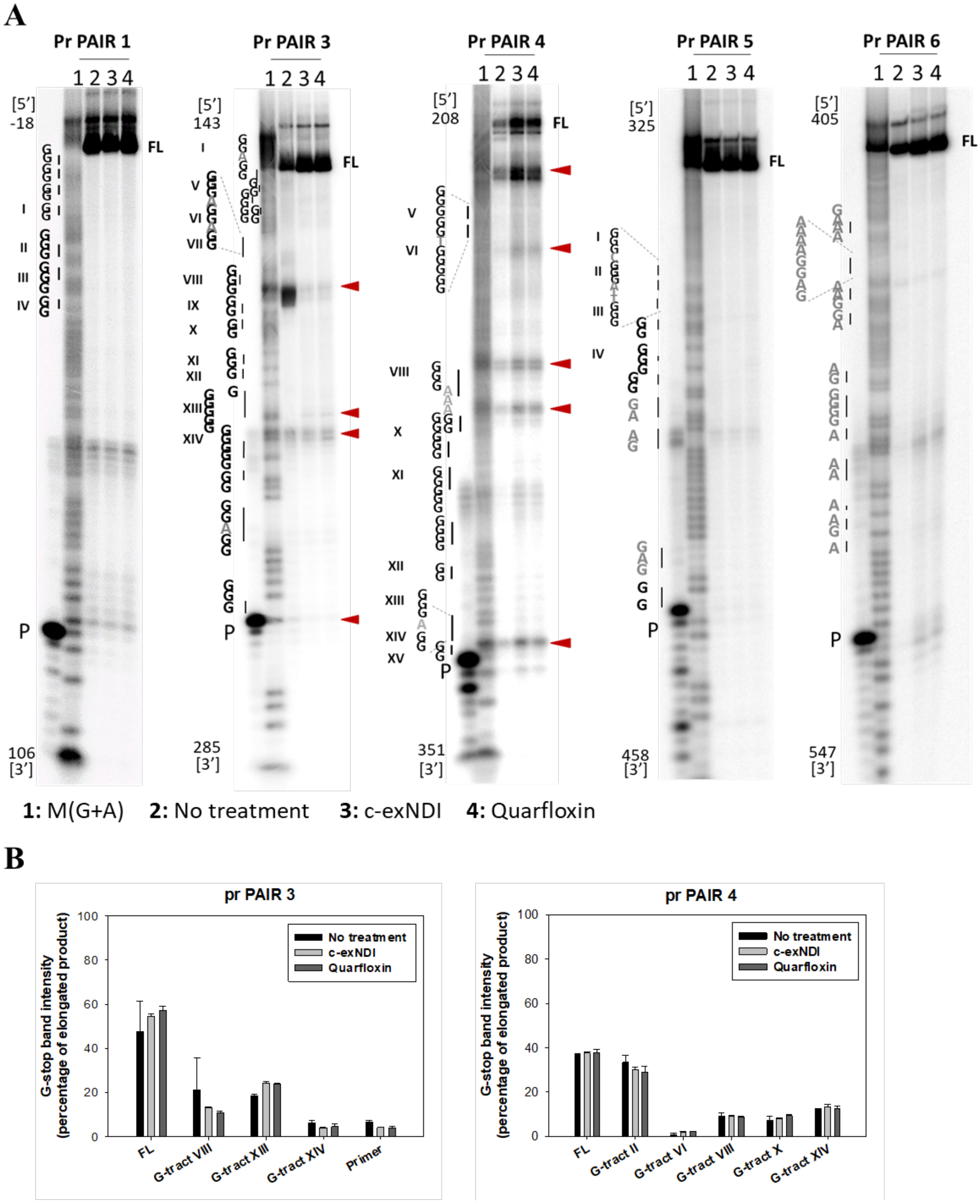

Supplementary Figure 1. PCR stop assay of *MDM2* P2 promoter. A) Amplification products obtained by PCR in the absence of G4-ligand (lane 2) or in the presence of 200 nM c-exNDI (lane3) and Quarfloxin (lane 4) with primers pairs 1, 3, 4, 5 and 6 are displayed. Lane 1 is the amplified regions reacted according to the Maxam and Gilbert protocol to visualize Gs and As. 'P' indicates a lane loaded with just the PCR radiolabelled primer and 'FL' refers to the full-length amplification product. The numbers on the left of each gel indicate the position of the amplified region with respect to the full-length *MDM2* P2 sequence. Gs involved in G4 formation

are reported in black and G-tracts are indicated with Roman numerals. Gray coloured bases are not involved in G4 formation and are reported to facilitate sequence reconstruction. The complete amplified sequence is reported in Supplementary Table 2. **B)** Quantification of lanes 2-4 of G4-compatible stops is shown in panel A. Quantification of stop bands observed in the *Mdm2* promoter regions amplified by primer pairs 3 and 4 and standard errors corresponding to the indicated PCR stops and of the full-length amplification product (FL) are reported as mean of two independent experiments.

Supplementary Table 2. Name and sequence of the oligonucleotides and primers used in this study. Oligonucleotides name, sequence and experiment in which they were employed is reported. Bold Gs are those involved in G4 formation. In the Mdm2-G4 mutants, the mutated bases are underlined and in red.

**Name**	**Sequence**
**WILD TYPE OLIGONUCLEOTIDES (CD, DMS, LC-MS)**	
Mdm2-G4	5' - T**GGGGG**CTC**GGGG**CGC**GGGG**CGC**GGGG**CAT**GGGG**C - 3'
Mdm2-G4-L	5' - **G**TTC**G**T**GG**CT**GGGGG**CTC**GGGG**CGC**GGGG**CGC**GGGG**CAT**GGGG**C - 3'
Mdm2-G4-1-4	5'-TGGGGGCTCGGGGCGCGGGGCGCGGGGCAT-3'
Mdm2-G4-2-5	5'-CTCGGGGCGCGGGGCGCGGGGCATGGGGC-3'
**SINGLE BASE MUTANTS**	
M1	5' - **G**TTC**G**T**GG**CT**GGGGG**CTC**GGGG**CGCT**GGG**CGC**GGGG**CAT**GGGG**C – 3'
M2	5' - **G**TTC**G**T**GG**CT**GGGGG**CTC**GGGG**CGC**G**T**GG**CGC**GGGG**CAT**GGGG**C – 3'
M3	**5' -** **G**TTC**G**T**GG**CT**GGGGG**CTC**GGGG**CGC**GG**T**G**CGC**GGGG**CAT**GGGG**C – 3'
M4	5' - **G**TTC**G**T**GG**CTT**GGGG**CTC**GGGG**CGC**GGGG**CGC**GGGG**CAT**GGGG**C – 3'
M5	5' - **G**TTC**G**T**GG**CT**GGGG**TCTC**GGGG**CGC**GGGG**CGC**GGGG**CAT**GGGG**C – 3'
M11	5' – **G**TTC**G**TT**G**CT**GGGGG**CTC**GGGG**CGC**GGGG**CGC**GGGG**CAT**GGGG**C - 3'
**TWO BASES MUTANTS**	
M6	5' – **G**TTC**G**T**GG**CT**G**T**G**T**G**CTC**GGGG**CGC**GGGG**CGC**GGGG**CAT**GGGG**C - 3'
M7	5' - **G**TTC**G**T**GG**CT**GGGGG**CTC**G**T**G**TCGC**GGGG**CGC**GGGG**CAT**GGGG**C - 3'
M8	5' - **G**TTC**G**T**GG**CT**GGGGG**CTC**GGGG**CGCT**G**T**G**CGC**GGGG**CAT**GGGG**C 3'
M9	5' - **G**TTC**G**T**GG**CT**GGGGG**CTC**GGGG**CGC**GGGG**CGCT**G**T**G**CAT**GGGG**C - 3'
M10	5' - **G**TTC**G**T**GG**CT**GGGGG**CTC**GGGG**CGC**GGGG**CGC**GGGG**CATT**G**T**G**C - 3'
M12	5' - **G**TTC**G**T**GG**CT**G GGGG**CTC**G**T**G**TCGC**GGGG**CGCT**G**T**G**CAT**GGGG**C - 3'
**PRIMERS (PCR stop assay)**	
Pr PAIR 1	FW: CCGGATGGTGAGGAGCAGG – RV: CGAACCGCACAAAGGCTGC
Pr PAIR 2	FW: CTGGTTCCCAGCCTCTGC – RV: CAAAACCTCCGCAAAGCC
Pr PAIR 3	FW: GGCACGTGGCTTTGC – RV: CGTGACCTTTACCCTGAACTCC
Pr PAIR 4	FW: GGATTTCGGACGGCTCTC – RV: GACCCGACAGGCACCTGC
Pr PAIR 5	FW: GGATGATCGCAGGTGCCTG – RV: GTTTCGGAACGTGTCTGAACTTG
Pr PAIR 6	FW: GGTTGACTCAGCTTTTCCTCTTG – RV: CTCCAATCGCCACTGAACACAG
**PRIMERS (ChIP-qPCR)**	
RPA3	FW: CGGAAGTTGACAGATACAGGG – RV: GATCGCAGAAAGGTAGTCTCAG
EIF4A	FW: CCGGAGCGACTAGGAACTAAC – RV: GCCTTTCTTACCGGGAATCCT
MDM2	FW: GGATTTCGGACGGCTCTCG - RV: CGTTCACACTAGTGACCCGA
TMCC2	FW: CCAGACACTTTGGGTGACCT – RV: AACACCTGCTCTGCCAACTT

NFASC	FW: CAGTGGGTTTGACCCCTAGA – RV: CACCTTCAGCTCCTCCTTTG
MAP3K13	FW: GACATAGGAACGGGCAAAGA – RV: CCCATGCTGTATGTGGTCTG
**TAQ-POL STOP ASSAY**	
primer	GGCAAAAAGCAGCTGCTTATATGCAG
Mdm2	TTTTTT**GGGGG**CTC**GGGG**CGC**GGGG**CGC**GGGG**CAT**GGGG**CTTTTTCTGCATATAAGCAGC
Non-G4 cnt	TTGTCGTTAAAGTCTGACTGCGAGCTCTCAGATCCTGCATATAAGCAGCTGCTTTTTGCC
**PRIMERS AND PROBES (RNA RT-PCR)**	
MDM2	FW: TGCAATACCAACATGTCTGTACCTA - RV: AGGGTCTCTTGTTCCGAAGC – Probe: 6FAM-TGATGGTGCTGTAACCACCTCACAGAT-TAMRA
GAPDH	FW: CCTCCCGCTTCGCTCTCT – RV: GCTGGCGACGCAAAAGA – Probe: 6FAM-CCTCCTGTTCGACAGTCAGCCGC-TAMRA
**BIOTINILATED OLIGONUCLEOTIDES (Protein pull-down)**	
Mdm2-G4-biot	5' - [BtnTg]T**GGGGG**CTC**GGGG**CGC**GGGG**CGC**GGGG**CAT**GGGG**C - 3'
G-ss-biot	5' - [Btn]TTTTTGGAGTCGTGTCGCGTGTCGAGCGTGTGTAGTGGTTTTT - 3'
C-ss-biot	5' - [Btn]AAAAACCCCAGTCCCGCCCAGGCCACGCCTCCCAAAAA - 3'

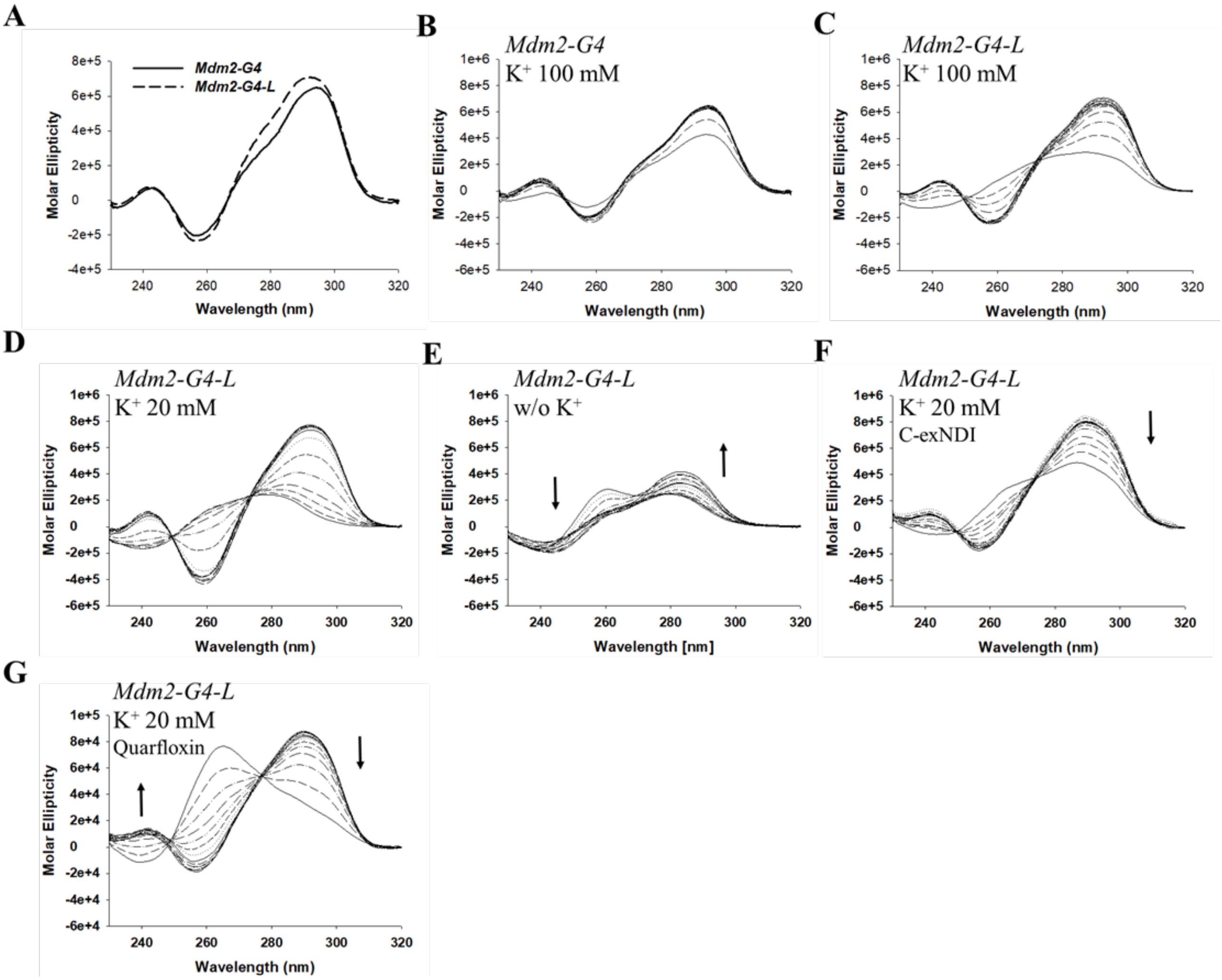

Supplementary Figure 2. CD thermal unfolding of *Mdm2-G4* and *Mdm2-G4-L* at different potassium concentrations and in the presence/absence of G4-ligands. A) CD spectra of *Mdm2-G4* and *Mdm2-G4-L* in the presence of 100 mM K^+ at 20 °C. **B)** Thermal unfolding of *Mdm2-G4* in the presence of K^+ 100 and **C)** of *Mdm2-G4 –L* in the presence of K^+ 100 mM; **D)** K^+ 20 mM; **E)** no K^+ **F)** K^+ 20 mM + c-exNDI; **G)** K^+ 20 mM + Quarfloxin.

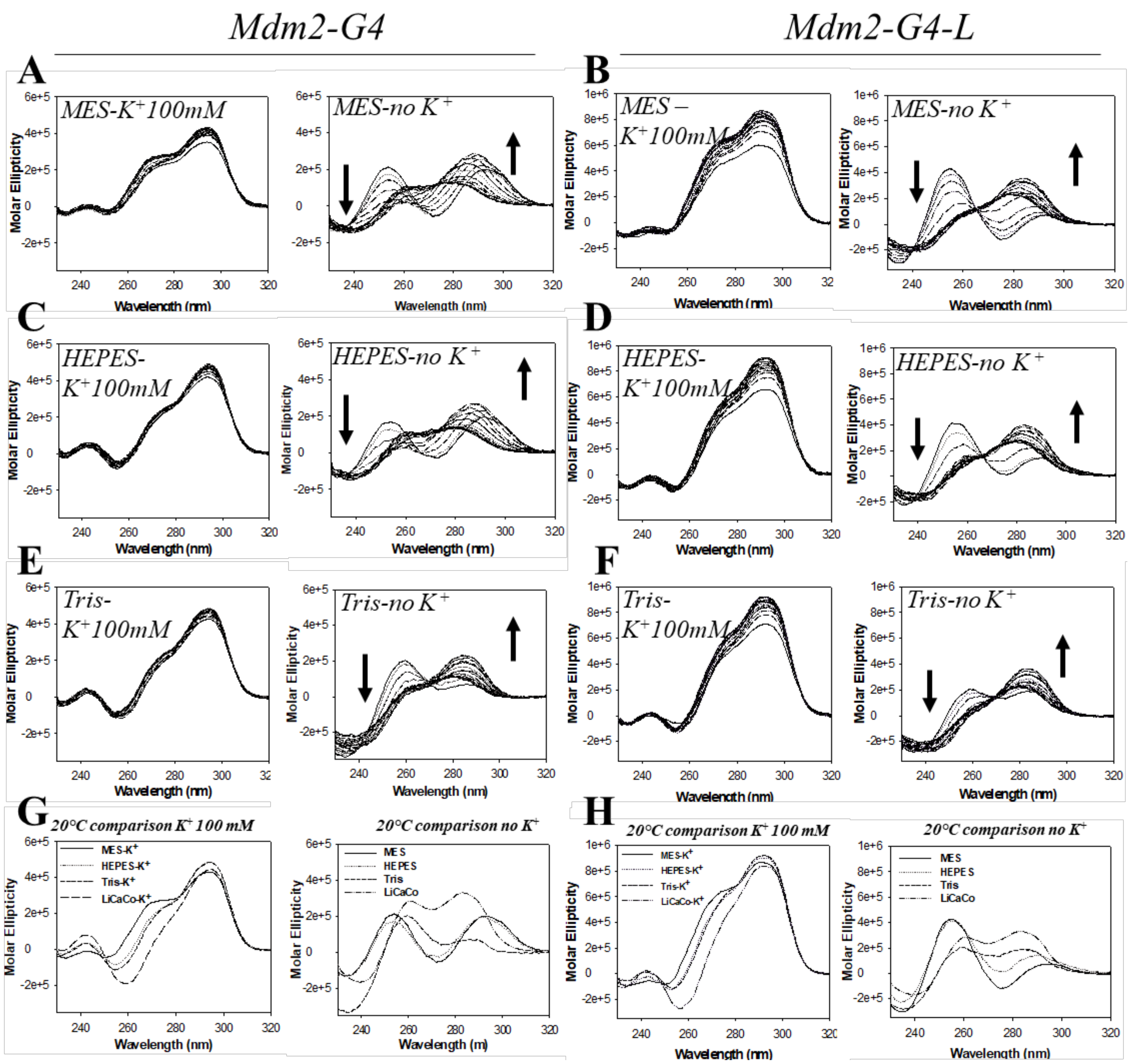

Supplementary Figure 3. CD analysis of K^+ stabilizing effect on *Mdm2-G4* and *Mdm2-G4-L* in the presence of different buffers. A, B) *Mdm2-G4* and *Mdm2-G4-L* in the presence or absence of K^+ 100mM in MES buffer. **C, D)** *Mdm2-G4* and *Mdm2-G4-L* in the presence or absence of K^+ 100mM in HEPES buffer. **E, F)** *Mdm2-G4* and *Mdm2-G4-L* in the presence or absence of K^+ 100mM in Tris buffer. **G, H)** Comparison of 20°C spectra of *Mdm2-G4* and *Mdm2-G4-L* in the presence or absence of K^+, in MES, HEPES, Tris and LiCaCo buffers.

Supplementary Table 3. CD melting temperatures of *Mdm2-G4* and *Mdm2-G4-L* in the presence or absence of 100 mM K^+ and different inert buffers. Delta T_{ms} calculated by CD spectroscopy in the presence of K^+ 100 mM. Standard errors are calculated from two independent replicates.

Oligo	***Buffer***	***K^+***	***Tm °C (260 nm)***	***Tm °C (290 nm)***	***Δ Tm °C (290 nm)***
Mdm2-G4	LiCaco	\	28.5 ± 1.1	54.3 ± 0.2	\
Mdm2-G4	MES	\	34.7 ± 1.0	57.6 ± 0.4	\
Mdm2-G4	HEPES	\	28.6 ± 0.9	50.1 ± 0.5	\
Mdm2-G4	Tris	\	32.5 ± 0.2	57.2 ± 0.6	\
Mdm2-G4	LiCaco	100 mM	\	86.3 ± 0.8	32 ± 1.0
Mdm2-G4	MES	100 mM	\	> 90	> 32.4
Mdm2-G4	HEPES	100 mM	\	> 90	> 39.9
Mdm2-G4	Tris	100 mM	\	> 90	> 32.8
Mdm2-G4-L	LiCaco	\	29.9 ± 1.3	55.1 ± 0.6	\
Mdm2-G4-L	MES	\	36.3 ± 0.7	61.4 ± 0.7	\
Mdm2-G4-L	HEPES	\	29.2 ± 0.8	52.1 ± 1.1	\
Mdm2-G4-L	Tris	\	31.1 ± 1.1	59.5 ± 0.7	\
Mdm2-G4-L	LiCaco	100 mM	\	> 90	> 34.9
Mdm2-G4-L	MES	100 mM	\	> 90	> 28.6
Mdm2-G4-L	HEPES	100 mM	\	> 90	> 37.9
Mdm2-G4-L	Tris	100 mM	\	> 90	> 30.5

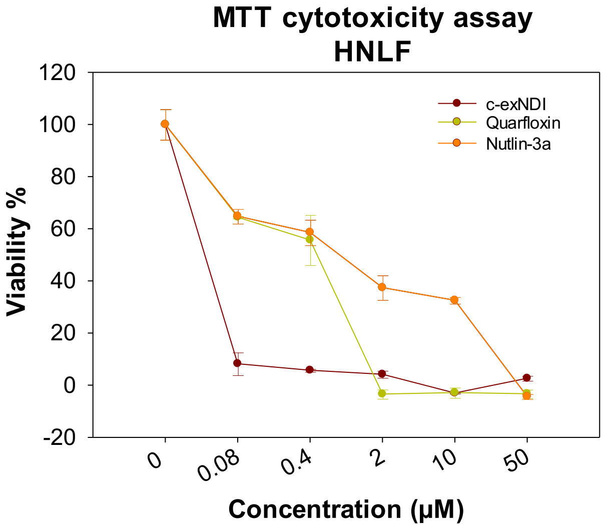

Supplementary Figure 4. MTT cytotoxicity assay on HNLF. Compound cytotoxicity was evaluated on HNLF upon 48 h treatment in the presence of c-exNDI, Quarfloxin and Nutlin-3a. Compounds were tested at the concentration range 0.08-50 µM. The resulting CC_{50} values are 0.004 ± 0.001 µM for c-exNDI, 0.503 ± 0.047 µM for Quarfloxin and 0.722 ± 0.067 µM for Nutlin-3a.

Supplementary Table 4. pG4s calculated in the human genome. Raw numbers of pG4s calculated in the whole human genome sequence (hg19, GRCh37) and on the OQs obtained by G4-seq (GEO accession GSE110582) are reported for each of the analysed pG4 category. OQ plus and OQ minus indicate the OQs identified on the positive and negative DNA strands. The detailed description of the pG4 categories is reported in the Material and Methods section.

	**OQ plus**	**OQ minus**	**OQ plus+minus**	**pG4s hg19**
**G3+L1–7 = canonical pG4s**	97039	96454	193493	361584
**G3+L1–12 = extended canonical pG4s**	132452	131291	263743	706337
**G4L1–7 = canonical four-tetrad pG4s**	11027	11113	22140	27385
**G4L1-12 = extended four-tetrad pG4s**	24515	24223	48738	63512
**G4L3 = *MDM2*-like pG4s**	604	639	1243	1707

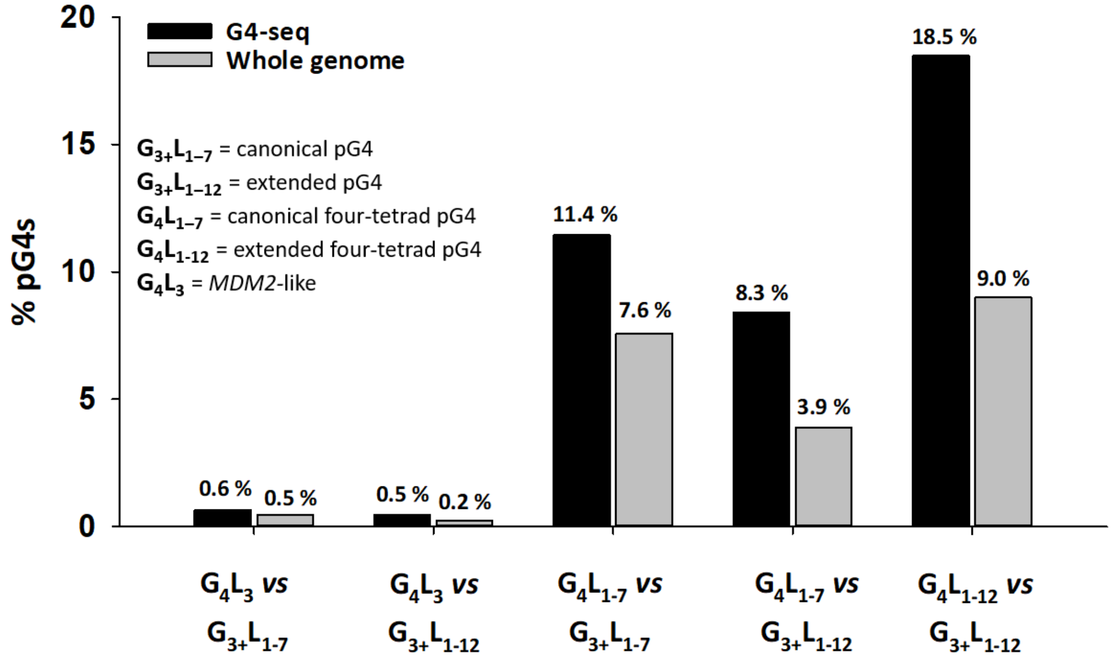

Supplementary Figure 5. Prediction of four-tetrad pG4s within the human genome. The percentage abundance of pG4s with four tetrads was calculated with Quadparser based on the whole human genome sequence (hg19, GRCh37 ‑ grey bars) and on the OQs obtained by G4-seq (GEO accession GSE110582 ‑ black bars). The five categories of analysed pG4s are reported in the figure legend.

Supplementary Table 5. Deconvolution of LC-MS mass spectra performed by mean of manual and automatic strategies. The theoretical oligonucleotide exact mass and oligonucleotide plus the indicated number of K^+ ions that it coordinates are shown along with the manually and software deconvoluted masses. The observed variation is always < 5 Da, which is within the expected error. The mass for a single K^+ used in the calculation is 39.0983 Da.

Oligo	Oligo theoretical MW (Da)	K^+ ions #	Oligo + K^+ MW (Da)	Manual deconvolution (Da)	Automatic deconvolution (Da)
Mdm2-G4	11043.807	3	11161.1019	11162.694	11163.4482
Mdm2-G4-L	13856.946	3	13974.2409	13970.012	13971.459
M12	13756.894	/	13756.894	13754.167	13750.042

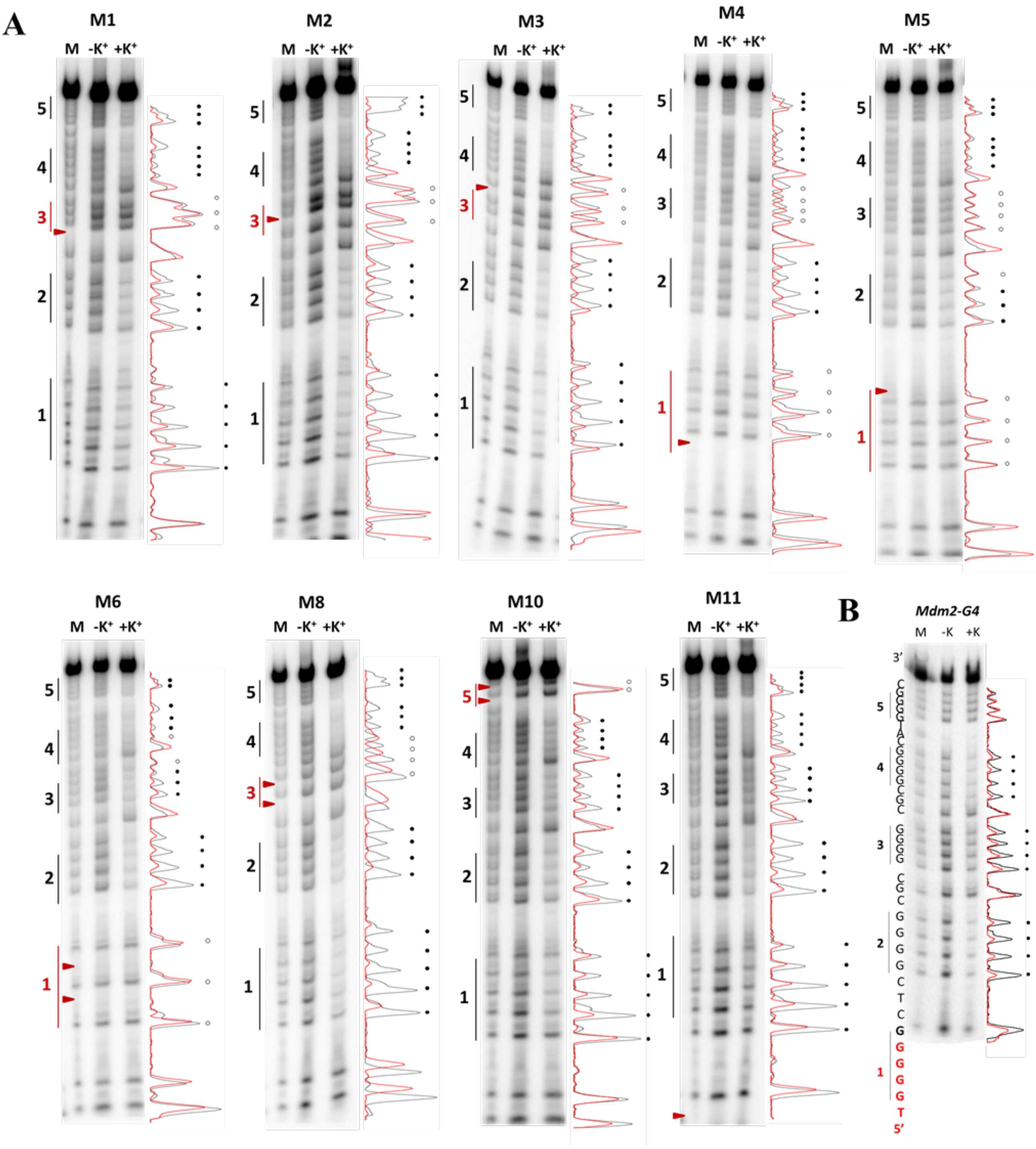

Supplementary Figure 6. DMS footprinting of *Mdm2-G4* mutants and *Mdm2-G4*. A) Mutants M1-6, 8, 10, 11**. B)** Mdm2-G4. In each gel M indicates lanes in which the oligonucleotide was treated according to the Maxam and Gilbert protocol to visualize G and A bases. -K^+ and +K^+ indicate the DMS reaction of the oligonucleotide in the absence and presence of K^+, respectively. Red triangles indicate the position of mutated bases, black circles the protected bases and white circles the bases that lost protection with respect to the wild type *Mdm2-G4L.*

Supplementary Table 6. CD melting temperatures of *Mdm2-G4* mutants in the presence of 100 mM K^+. Delta T_{ms} calculated by CD spectroscopy in the presence of K^+ 100 mM. Standard errors are calculated from two independent replicates.

Name	T_m mean (°C) 290 nm	ΔT_m (°C)
wt Mdm2-G4	> 90 °C	/
M1	79.3 ± 0.3	> 10.7 ± 0.3
M2	78.6 ± 0.3	> 11.4 ± 0.3
M3	78.8 ± 0.5	> 11.2 ± 0.5
M4	87.8 ± 1.9	> 2.2 ± 1.8
M5	> 90 °C	/
M6	> 90 °C	/
M7	86.3 ± 2.8	> 3.7 ± 2.8
M8	78.1 ± 0.8	> 11.9 ± 0.7
M9	89.6 ± 3.2 °C	/
M10	85.3 ± 0.4	> 4.7 ± 0.4
M11	> 90 °C	/
M12	68.0 ± 1.2	> 22.0 ± 1.2

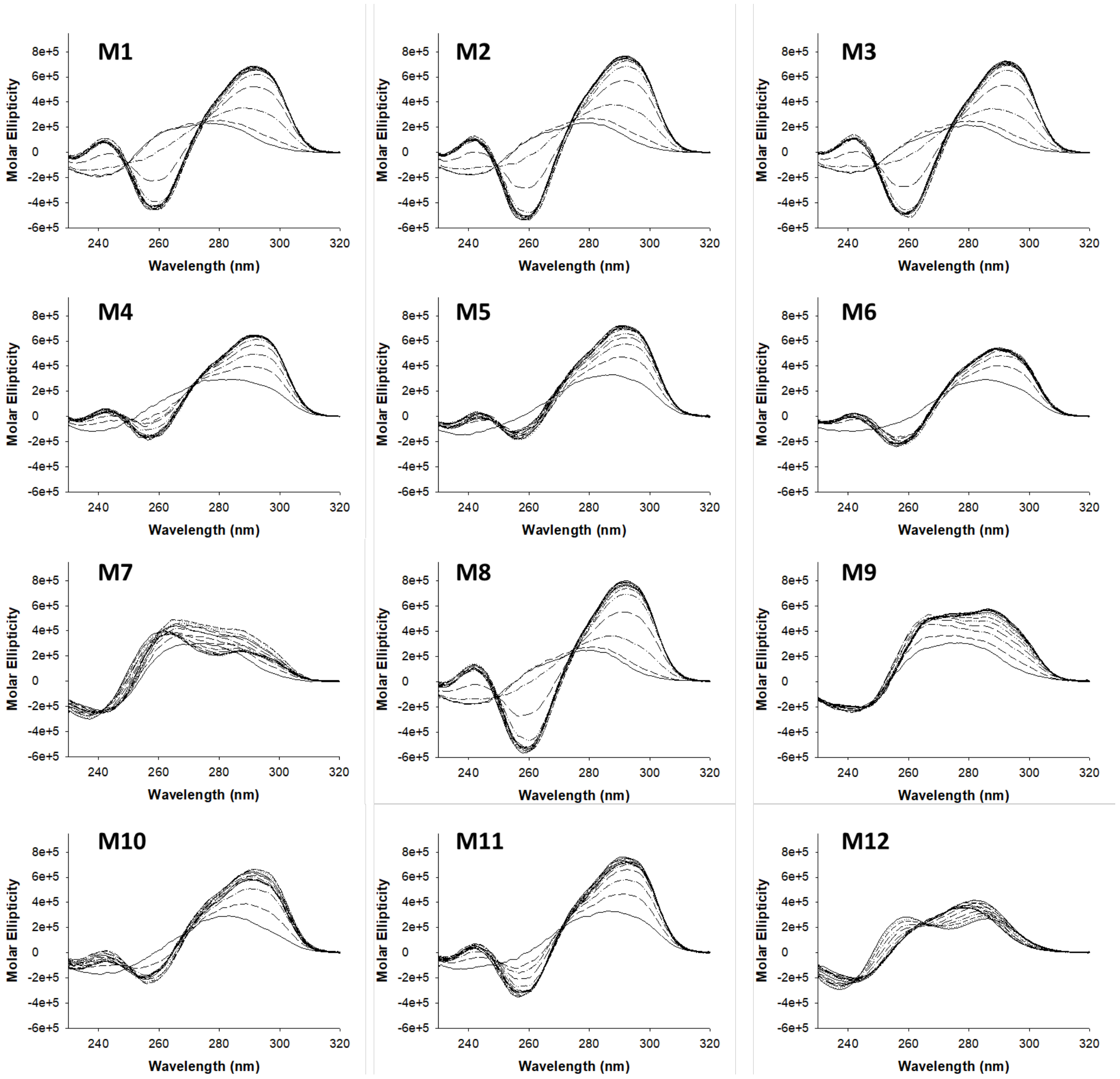

Supplementary Figure 7. CD thermal unfolding spectra of *Mdm2-G4* mutants. Spectra were recorded over the temperature range 20-90 °C in the presence of 100 mM K^{+}. The mutants' name is reported in the upper right part of each panel. The oligonucleotides' sequences are reported in Supplementary Table 1.

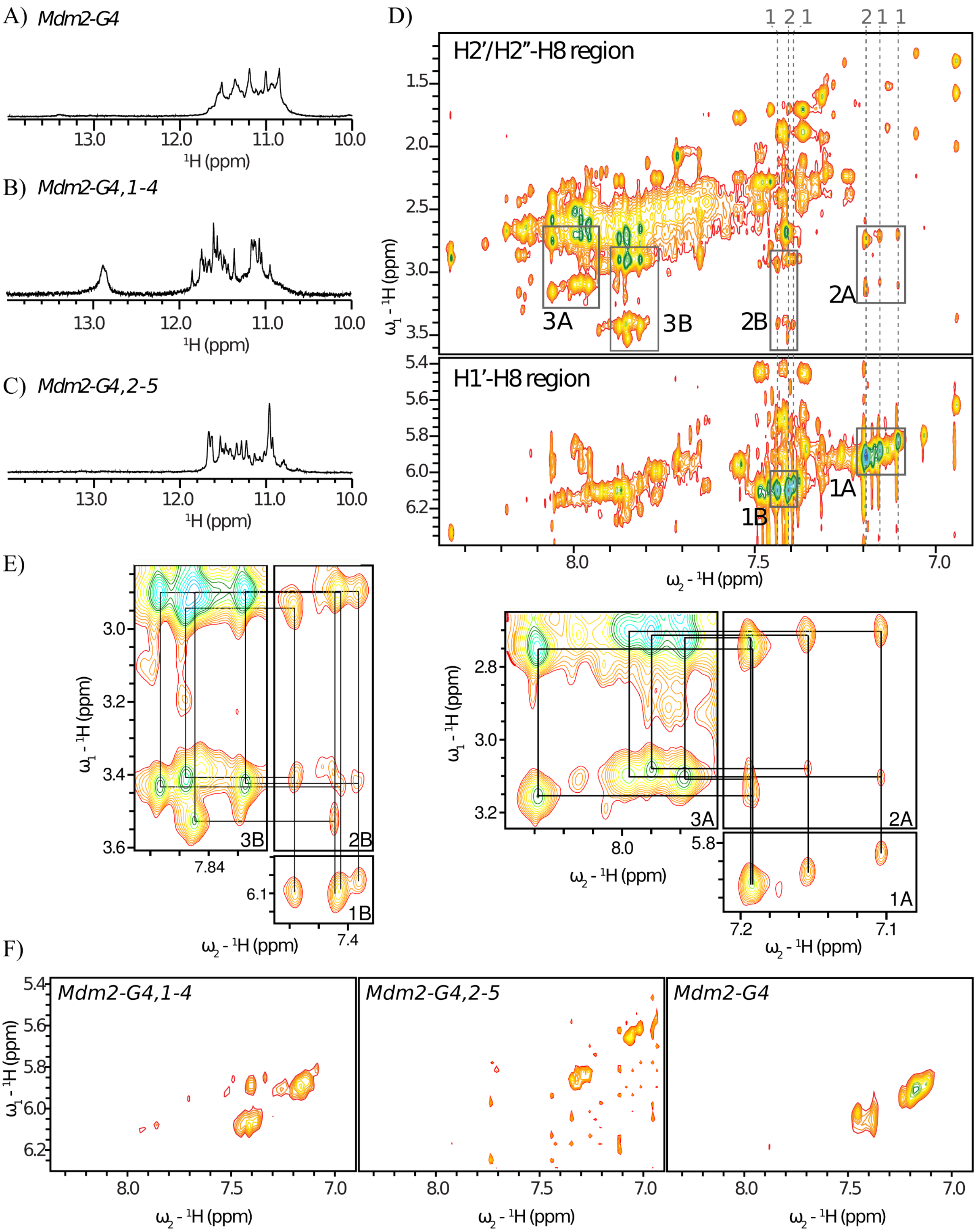

Supplementary Figure 8. NMR characterization of *Mdm2-G4*, *Mdm2-G4,1-4* and *Mdm2-G4,1-5* oligonucleotides. Imino region of 1D ^{1}H spectrum of **A)** *Mdm2-G4*, **B)** *Mdm2-G4,1-4* and **C)** *Mdm2-G4,2-5* in the presence of 100 mM K$^+$. **D)** H1'-H8 and H2'/H2''-H8 region of 2D NOESY spectrum (τ_m = 200 ms) of *Mdm2-G4,1-4*. Rectangles labelled 1A and 1B show two groups of four strong H1'-H8 cross-peaks of guanine

residues in *syn* conformation along glycosidic bond, while rectangles labelled 2A and 2B show characteristic weak H2'/H2''-H8 cross-peaks of the same guanine residues in *syn* conformation with down-field shifted H2'/H2'' resonances. Rectangles labelled 3A and 3B show two groups of residues in *anti* conformation involved in sequential steps with residues in *syn* conformation. Numbers on the top of the spectrum denote number of guanine residues that give rise to cross-peaks in 2D NOESY spectrum. **E)** Magnification of regions labelled with rectangles in D) with vertical lines connecting intra-residual cross-peaks along aromatic proton and horizontal lines connecting sequential cross-peaks along sugar proton chemical shifts. Vertical intensity of regions 1A and 1B is decreased by ten times compared to 2A, 3A, 2B and 3B. **F)** H1'-H8 region of 2D NOESY spectra (τ_m = 80 ms) of *Mdm2-G4,1-4*, *Mdm2-G4,2-5* and *Mdm2-G4*.

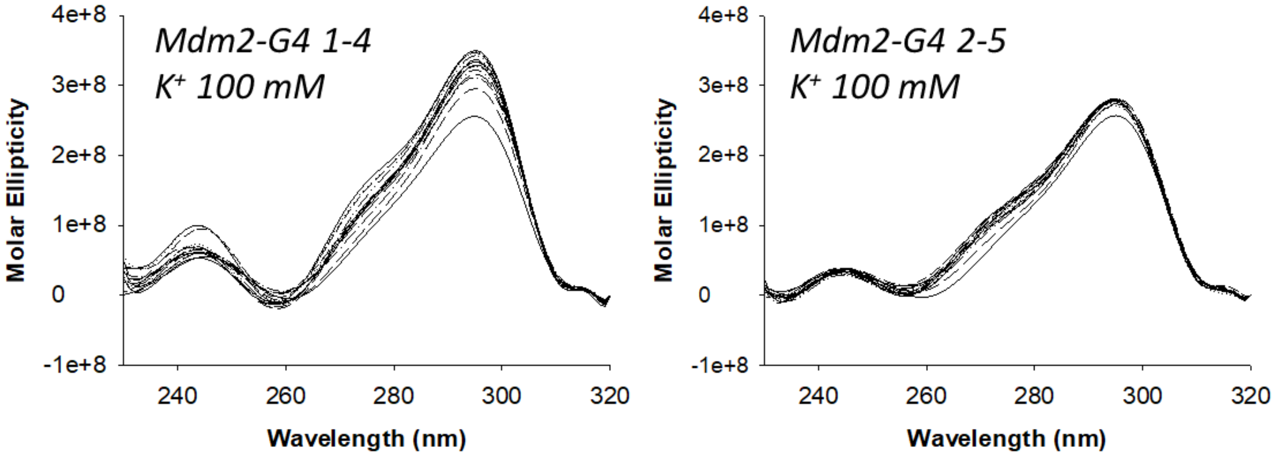

Supplementary Figure 9. CD spectra of Mdm2-G4, 1-4 and Mdm2-G4, 2-5. Spectra of oligonucleotides Mdm2-G4, 1-4 (left) and Mdm2-G4, 2-5 (right), corresponding to short versions of *Mdm2-G4* oligonucleotide in which the fifth and first G-tracts respectively were eliminated. Spectra were recorded in the presence of 100 mM K^+.

Supplementary Methods and Materials

In the following section supplementary material and methods of the most standard techniques that were employed in the realization of the project are reported.

MTT cytotoxicity assay

Cytotoxicity of c-exNDI and Nutlin-3a in 93T449 cells was determined by MTT assay. 7500 cells/well were plated in a 96-wells plate and grown for 24 h. Serial dilutions of the compounds (0.08–50 μM) were dispensed to the cells in triplicate. After 48 h from treatment, cells were supplemented with freshly diluted 3-(4,5-dimethylthiazol-2-yl)-2,5-diphenyltetrazolium bromide (MTT) (Sigma-Aldrich, Milan, Italy) solution (5 mg/mL) and incubated O/N at 37 °C. MTT crystals were solubilized with the addition of 100 μl solubilization solution (10 % SDS and 0.01 M HCl) for 8 h at 37 °C and absorbance was measured in Sunrise Tecan plate reader (Mannendorf, Switzerland) at 620 nm. The percentage of cell survival was calculated as follows: cell survival = $(A_{well} − A_{blank})/(A_{control} −A_{blank}) \times 100$, where “blank” denotes the medium without cells and “control” the untreated cells. The 50% cytotoxic concentration (CC_{50}) was defined as the concentration of compound required to reduce cell growth by 50%.

Real time PCR

93T449 cells were seeded in 6-well and treated with c-exNDI, Quarfloxin or DMSO as indicated. Total RNA was isolated using TRIzol reagent (Life Technologies, Monza, Italy) according to the manufacturer’s instructions and subjected to RNase free DNase I treatment (Ambion Turbo DNA free, Life Technologies, Monza, Italy). Extracted RNA was subjected to One-Step (Ambion™, cat. AM1005) reverse transcription and Real-time PCR using TaqMan chemistry with 5′-[FAM] and 3′-[TAMRA] end labeled probes (**Supplementary Table 1**). Experiments were performed using ABI 7900 HT – FAST Real time PCR System under the following conditions: 30 min at 50 °C for reverse transcription, 95 °C for 10 min followed by 40 cycles of 30 seconds at 95 °C and 30 seconds at 58 °C, 1 minute at 72 °C. mRNA transcription levels were standardized against the housekeeping gene GAPDH. Non-treated retrotranscribed RNA collected right before of G4-ligand or DMSO treatment was used as mRNA expressed control. The experiment was performed in triplicate and within each experiment samples were analysed in duplicate.

Western Blot

93T449 cells were seeded in 6-well plates, grown overnight and then treated with c-exNDI, Quarfloxin or DMSO as indicated. Cells were lysed in RIPA buffer (NaCl 150 mM, IGEPAL 1%, SDS 0.1%, Tris-HCl pH 7.5 50 mM, Na-deoxycolate 0.5 % + protease inhibitors cocktail). Protein concentration was quantified using the Pierce® BCA Protein Assay Kit (Thermo Scientific, Rockford, IL, USA), electrophoresed on 10% SDS-PAGE and transferred to aPVDF blotting membrane (Merck,,Italy) The membranes were blocked with 2.5% skim milk in PBS. Membranes were incubated with the respective primary antibody directed against MDM2 (SMP14 sc-965, Santa Cruz Biotechnology, Dallas, TX, USA), hnRNP-K (D-6 sc-28380, Santa Cruz Biotechnology, Dallas, TX, USA), p53 (DO-1 sc-126, Santa Cruz Biotechnology, Dallas, TX, USA) and β-actin (mouse monoclonal; Sigma-Aldrich, Milan, Italy). After three washes in PBST (0.05 % Tween 20 in PBS), membranes were

incubated with ECL Plex HRP-Goat-α-Mouse IgG (Millipore). Images were captured on an Uvitec bioluminescence reader.

Flow cytometry

93T449 cells were seeded in 6-well plates, grown overnight and then treated with c-exNDI or Quarfloxin for 24, 48 or 72 h as specified. For the evaluation of cell cycle arrest cells were fixed in the presence of 70% ethanol for 15 min at 4 °C and subjected to RNA digestion with 5 μl DNase-free RNAse A [10 mg/ml] (Thermo Scientific, cat. EN0531) for 30 min at 37 °C. Right before FACS analysis 10 μl of Propidium iodine (PI) [1 mg/ml] were added to each sample. Apoptosis was evaluated using the Annexin V-FITC Apoptosis detection kit (eBioscience, ThermoScientific) according to the manufacturer instruction. Data analysis was performed with Flowing Software 2 (http://www.flowingsoftware.com) (22).

Circular Dichroism

For CD spectroscopy analysis, oligonucleotides were diluted to a final concentration of 4 μM in lithium cacodylate buffer Lithium Cacodylate (10 mM, pH 7.4), MES (10 mM, pH 7.4), HEPES (10 mM, pH 7.4) or Tris (10 mM, pH 7.4) with or without KCl at the concentration indicated in text. After heat-denaturation at 95 °C for 5 minutes, samples were folded at room temperature overnight. For experiment in the presence of G4-ligands, the compound at concentration of 16 μM was added 4 h after oligonucleotide denaturation. CD spectra were recorded on a Chirascan-Plus (applied Photophysocs, Leatherhead, UK) equipped with a Peltier temperature controller using a quartz cell of 5 mm optical path length. For T_m determination, spectra were recorded over a temperature range of 20-90 °C, with temperature increase of 5 °C/min over a wavelength range of 230-320 nm. T_m values were calculated according to the van't Hoff equation, applied for a two-state transition from a folded to unfolded state. Acquired spectra were baseline corrected for signal contribution due to the buffer and the observed ellipticities converted to mean residue ellipticity (θ) = deg x cm^2 x $dmol^{-1}$ (molar ellipticity).
